# A Comprehensive Evaluation of Sdox, a Promising H_2_S-Releasing Doxorubicin for the Treatment of Chemoresistant Tumors

**DOI:** 10.3389/fphar.2022.831791

**Published:** 2022-03-07

**Authors:** Petko Alov, Merilin Al Sharif, Denitsa Aluani, Konstantin Chegaev, Jelena Dinic, Aleksandra Divac Rankov, Miguel X. Fernandes, Fabio Fusi, Alfonso T. García-Sosa, Risto Juvonen, Magdalena Kondeva-Burdina, José M. Padrón, Ilza Pajeva, Tania Pencheva, Adrián Puerta, Hannu Raunio, Chiara Riganti, Ivanka Tsakovska, Virginia Tzankova, Yordan Yordanov, Simona Saponara

**Affiliations:** ^1^ Department of QSAR and Molecular Modelling, Institute of Biophysics and Biomedical Engineering, Bulgarian Academy of Sciences, Sofia, Bulgaria; ^2^ Department of Pharmacology, Pharmacotherapy and Toxicology, Faculty of Pharmacy, Medical University of Sofia, Sofia, Bulgaria; ^3^ Department of Drug Science and Technology, University of Torino, Torino, Italy; ^4^ Department of Neurobiology, Institute for Biological Research Siniša Stanković, National Institute of Republic of Serbia, University of Belgrade, Belgrade, Serbia; ^5^ Institute of Molecular Genetics and Genetic Engineering, University of Belgrade, Belgrade, Serbia; ^6^ BioLab, Instituto Universitario de Bio-Orgánica Antonio González, Universidad de La Laguna, La Laguna, Spain; ^7^ Department of Biotechnology, Chemistry and Pharmacy, University of Siena, Siena, Italy; ^8^ Institute of Chemistry, University of Tartu, Tartu, Estonia; ^9^ School of Pharmacy, University of Eastern Finland, Kuopio, Finland; ^10^ Department of Oncology, University of Torino, Torino, Italy; ^11^ Department of Life Sciences, University of Siena, Siena, Italy

**Keywords:** cytochrome P450, doxorubicin, hepatotoxicity, hERG, *in silico* profiling, off-targets, P-glycoprotein, zebrafish

## Abstract

Sdox is a hydrogen sulfide (H_2_S)-releasing doxorubicin effective in P-glycoprotein-overexpressing/doxorubicin-resistant tumor models and not cytotoxic, as the parental drug, in H9c2 cardiomyocytes. The aim of this study was the assessment of Sdox drug-like features and its absorption, distribution, metabolism, and excretion (ADME)/toxicity properties, by a multi- and transdisciplinary *in silico*, *in vitro*, and *in vivo* approach. Doxorubicin was used as the reference compound. The *in silico* profiling suggested that Sdox possesses higher lipophilicity and lower solubility compared to doxorubicin, and the off-targets prediction revealed relevant differences between Dox and Sdox towards several cancer targets, suggesting different toxicological profiles. *In vitro* data showed that Sdox is a substrate with lower affinity for P-glycoprotein, less hepatotoxic, and causes less oxidative damage than doxorubicin. Both anthracyclines inhibited CYP3A4, but not hERG currents. Unlike doxorubicin, the percentage of zebrafish live embryos at 72 hpf was not affected by Sdox treatment. In conclusion, these findings demonstrate that Sdox displays a more favorable drug-like ADME/toxicity profile than doxorubicin, different selectivity towards cancer targets, along with a greater preclinical efficacy in resistant tumors. Therefore, Sdox represents a prototype of innovative anthracyclines, worthy of further investigations in clinical settings.

## Introduction

A library of hydrogen sulfide (H_2_S)-releasing doxorubicin analogues was synthesized to overcome the well-known cardiotoxicity (Sawicki et al., 2021) and the drug resistance (Assaraf et al., 2019) characterizing doxorubicin (Dox) treatment and limiting its use in cancer therapy (Chegaev et al., 2016). A series of derivatives, devoid of toxicity in H9c2 cardiomyocytes, but still retaining their efficacy in U-2OS osteosarcoma cells, characterized by increasing levels of P-glycoprotein (P-gp) and resistance to Dox, have been synthesized (Chegaev et al., 2016).

Sdox, for example, displayed cytotoxic activity *in vitro* in an androgen-independent and Dox-resistant DU-145 prostate cancer cell line and reduced tumor mass volume in a castration-resistant prostate cancer xenograft model (Bigagli et al., 2018). In U-2OS and Saos-2-chemoresistant osteosarcoma cells, Sdox, unlike Dox, accumulated within the endoplasmic reticulum (ER), where it releases H_2_S that sulfhydrated nascent proteins, including P-gp, increasing their misfolding and triggering ER-dependent apoptosis (Buondonno et al., 2019). This process enhanced both retention and toxicity of Sdox in resistant cells.

Sdox and, to a greater extent, Sdox in a liposomal formulation decorated with hyaluronic acid (HA-Lsdox) reduced the growth of osteosarcoma refractory to both Dox and Caelyx^®^, the pegylated liposomal Dox, currently used in clinical setting (Gazzano et al., 2019).

Additionally, Sdox was not cytotoxic as Dox in H9C2 cardiomyocytes (Chegaev et al., 2016; Buondonno et al., 2019) and displayed the same cardiotoxicity profile of Caelyx^®^ in osteosarcoma xenograft (Gazzano et al., 2019).

Elevated liver enzymes and acute liver injury with jaundice, which can be serious and even fatal, have been reported during Dox treatment (LiverTox, 2018; Prasanna et al., 2020). After all, Dox is eliminated in large part by the liver, and several mechanisms of Dox-induced hepatotoxicity have been proposed, including free radical formation and mitochondrial dysfunction (Kalender et al., 2005; Prasanna et al., 2020). Abnormal liver blood test result is a rare side effect reported also in Caelyx^®^ summary of product characteristics (European Medicines Agency, 2021).

Therefore, Dox and Caelyx^®^ dosage should be adjusted in patients with hepatic impairment (Food and Drug Administration, 2013).

Several strategies have been attempted to overcome MDR and either prevent or alleviate these Dox unwanted effects.

Although Sdox may represent a lead compound for the treatment of chemoresistant tumors, its clinical development might be limited by severe adverse reactions that characterize the parent compound (Food and Drug Administration, 2013). Moreover, nonclinical safety-related attrition represents a major issue for research and development of novel drugs, particularly during lead identification and optimization stages (Blomme and Will, 2016).

The principal side effects of a drug originate from (1) chemical-based effects, related to its physicochemical features; (2) on-target, mechanism-based effects; and (3) off-target effects, due to binding to unintended targets (e.g., proteins or nucleic acids) (Rudmann, 2013).

Therefore, the aim of this study was the *in silico* as well as *in vitro* and *in vivo* evaluation of Sdox drug-like characteristics, and anti-cancer efficacy, absorption, distribution, metabolism, and excretion/toxicity (ADME/Tox) properties, using Dox as a reference compound.

This multidisciplinary work was performed by a group of researchers from COST Action STRATAGEM “New diagnostic and therapeutic tools against multidrug resistant tumors.”

## Materials and Methods

### *In Silico* Drug Profiling

The ACD/Percepta software (version 2020.2, Advanced Chemistry Development, Inc., Toronto, ON, Canada) was initially used for Dox and Sdox profiling.

The individual predictions were categorized according to predefined thresholds for the following groups of parameters: (1) PhysChem (logP, solubility, H-bonding, molecular size and flexibility, drug and lead-likeness rules); (2) ADME [human intestinal absorption (HIA) and Caco-2 permeability, plasma protein binding (PBI), blood–brain barrier penetration (BBB)]; (3) drug safety [P-gp substrate, cytochrome P450 (CYP450) inhibitor, and hERG inhibitor specificity].

Categorization was based on either continuous properties (numerical thresholds set on the scale of the corresponding property values) or probabilistic predictors (for drug safety). Each prediction was assigned a score according to the equation: Score = (p—0.5) * RI + 0.5, where p is the obtained probability, and RI is the reliability index value. The score relies on the assumption that predicted probability close to 0.5 and low RI are both indicators of an inconclusive prediction. The term (p—0.5) * RI considers predictions where low RI values would ultimately yield scores in the intermediate range even if the original *p*-value is quite high or low. The final category assignment was based on the default classification score ranges.

The partition coefficient logP was calculated using two different predictive algorithms: ACD/LogP Classic (fragment-based approach) and ACD/LogP GALAS (similarity-based approach). The consensus logP was calculated as a weighted average of both predictions by assigning dynamic adaptive coefficients: each model obtained larger weight in those regions of chemical space where it performed most reliably. The models were as follows: for Dox (0.12 Classic+ 0.88 GALAS) and for Sdox (0.4 Classic +0.6 GA-LAS). In addition, the logP values of the compounds were estimated by Molecular Operating Environment (MOE) software (version 2019.01, Chemical Computing Group, ULC, Montreal, QC, Canada) and by Marvin (version 14.8.25, ChemAxon, Budapest, Hungary). In MOE, the “h_logP” descriptor was calculated by a multiparameter model built on 1,836 molecules [*r* ^2^ = 0.84, root mean square error (RMSE) = 0.59]. In Marvin, the logP values were calculated by an atom-based approach (Hansch et al., 1995).

The distribution coefficients (logD) of Dox and Sdox at pH = 7.0 were also calculated using ACD/Labs.

The Derek Nexus expert system (version 6.0.1, Lhasa Ltd., Leeds, United Kingdom) was used for toxicity prediction. The algorithm relies on the concept of “structural alerts” (toxicophores) defined as a set of structural features that makes a user suspect that the molecule may exert a particular effect. It compares the structural features of the query compound to the structural alerts in the knowledge database of the program. Prediction is based on a reasoning scheme, which considers the presence of a set of toxicophores in the query structure (Sutter et al., 2013). Seven likelihood levels were used as follows (in highest to lowest order of likelihood): certain—there is proof that the proposition is true; probable—there is at least one strong argument that the proposition is true and there are no arguments against it; plausible (baseline)—the level of likelihood indicating the weight of evidence supports the proposition; equivocal—there is an equal weight of evidence for and against the proposition; doubted—the weight of evidence opposes the proposition; improbable—there is at least one strong argument that the proposition is false and there are no arguments that it is true; and impossible—there is proof that the proposition is false (Judson et al., 2013). The following settings were applied: restrict to mammal species; perceive tautomers (yes); reasoning level threshold (plausible); and show negative predictions (yes).

### Metabolism Predictions

Metabolic transformations were predicted by the knowledge-based expert system Meteor Nexus (version 3.1.0, Meteor KB 2018 1.0.0, Lhasa Ltd., Leeds, United Kingdom).

Since reactive metabolites are generally produced by Phase I reactions (Njuguna et al., 2012), only those were considered in this study. The system allows for analysis of the assigned likelihood levels by either considering the relevance of each metabolite (absolute likelihood) or taking into account the likelihood of occurrence of a preceding metabolite as a factor affecting the appearance of metabolites resulting in the following steps (on path likelihood). The absolute reasoning (AR) method was used with the following default parameters: maximal depth (number of metabolic steps) = 3 and maximal number of metabolites = 1,000. The AR method relies on a similar reasoning scheme as toxicity predictions by Derek (see above) operating with five likelihood levels (probable, plausible, equivocal, doubted, and improbable) for a biotransformation to occur (Judson et al., 2013). The minimal likelihood level was set to consider only probable and plausible predictions.

### Docking and Binding to Pregnane-X-Receptor and Sulfotransferase

The protein structures of the pregnane-X-receptor (PXR) and sulfotransferase (SULT) were searched in the Protein DataBank (PDB) (Berman et al., 2000). Crystal structures and optimization protocols were as described previously (Glisic et al., 2016; Viira et al., 2016; Garcia-Sosa et al., 2009). Briefly, the crystal structures 1M13.pdb and 2A3R.pdb were downloaded, hydrogens added, and preprocessed with Maestro (Release 2021-4, Schrödinger LLC, New York, NY, United States). Thresholds for docked compounds were based on previously known binders (Glisic et al., 2016; Viira et al., 2016; Garcia-Sosa et al., 2009) and docking scores color-coded black, gray, or white, depending on closeness to the determined threshold.

Dox, Sdox, and their metabolites were drawn, hydrogens added, and geometry-optimized with Maestro (Schrödinger Release 2021-4, LLC, New York, NY, United States). Ligprep (Release 2021-4, Schrödinger LLC, New York, NY, United States) calculated tautomers, ionization states, and conformers for the metabolite structures using a pH of 7.4 with threshold of 2 units.

Docking was carried out using programs Glide XP (Schrödinger Release 2021-4, LLC, New York, NY, United States) and AutoDock (Morris et al., 2009), in addition to Vina (Trott and Olson, 2010). Each docking program uses a different algorithm for pose prediction and scoring in order to hedge values and increase confidence in the results. The best score was recorded for each different species of the same ligand. AutoDock 4 and Vina calculations were performed with 100 runs per ligand. Settings were default except for those previously described in (Glisic et al., 2016; Viira et al., 2016; Garcia-Sosa et al., 2009).

### Off-Target Prediction

Interactions of Dox and Sdox with 85 different targets were analyzed by computational docking using MOE software (version 2019.01, Chemical Computing Group, ULC, Montreal, QC, Canada). For *β*-tubulin, interactions at both the colchicine binding site (CBS) and the Vinca binding site (VBS) were investigated. Crystallographic structure of targets was obtained from PDB (list of PDB target IDs available in Supplementary Information) (Berman et al., 2000). Protein structures were energetically minimized using Amber10 force field with EHT parameters for small molecules, R-field solvation model, and dielectric constant of 1 for the protein interior and 80 for exterior. Dox and Sdox structures were drawn in MOE software (version 2019.01, Chemical Computing Group, ULC, Montreal, QC, Canada), and their energies were minimized according to the above parameters using as stop criterion an RMS gradient lower than 0.01 kcal/mol/Å. For docking calculations, the Triangle Matcher algorithm with the London dG scoring function in the placement stage was used. The receptor was rigid, and the GBVI/WSA dG scoring function was used in the refinement stage.

Results were expressed as docking score (DS) values and difference in DS (ΔDS) values obtained for Dox and Sdox against the same target in order to provide more relevant information in their mechanism of action (Palmeira et al., 2012).

### *In Vitro* P-gp Assay

To study the effect of P-gp overexpression in the antiproliferative activity of Dox and Sdox, a cell-line-based assay was performed by using wild-type cell line (SW1573) and its P-gp-overexpressing variant (SW1573/P-gp) (Baas et al., 1990). Compounds were tested against both cell lines in the presence or absence of 10 µM verapamil (a known P-gp and CYP3A4/5 inhibitor). The GI_50_ (the concentration that causes 50% growth inhibition) (Skehan et al., 1990) values after 48 h of exposure to drugs in absence (w/o) or presence (w) of verapamil were determined in both cell types. The microtubule-interacting drugs paclitaxel (known substrate of P-gp), colchicine, vincristine, and vinblastine were selected as reference compounds for comparison purposes (Baas et al., 1990).

### *In Vitro* Inhibition of Human CYP3A4

Dox and Sdox were tested *in vitro* for their ability to inhibit CYP3A4 enzyme recombinant human CYP3A4 (BD Biosciences Discovery Labware, Woburn, MA, United States). 3-(3-Benzyloxo)phenyl-7-methoxycoumarin was used as the substrate, the well-known CYP3A4 inhibitor ketoconazole as positive control, while negative controls were performed in the absence of substrate or enzyme. Kinetic assays were carried out in a final volume of 100 µl containing 10 µM substrate, 2.5 nM recombinant CYP3A4, various concentrations of the tested compounds, and 20% NADPH regenerating system in 100 mM Tris–HCl (pH 7.4). Incubations took place in duplicate at 37°C in 96-multiwell plates; fluorescence was measured with a Victor2 plate reader (PerkinElmer Life Sciences, Turku, Finland). The reaction was started by adding NADPH, and fluorescence was measured at 2-min intervals for 40 min using excitation at 405 nm and emission at 460 nm. IC_50_ (the concentration causing a 50% reduction of maximal activity) values were calculated using the equation v_i_/v_0_ = 1/(1 + I/IC_50_), where v_i_ is the rate at a fixed concentration of inhibitor, v_0_ is the rate recorded in the absence of the inhibitor (100% rate), and I is the inhibitor concentration (Juvonen et al., 2020).

### *In Vitro* Hepatotoxicity Studies

Human hepatoma (HepG2) cells (European Collection of Authenticated Cell Cultures) were cultured in Dulbecco’s modified Eagle’s medium (DMEM) supplemented with 10% heat-inactivated fetal bovine serum (FBS) and 2 mM L-glutamine at 37°C in 5% CO_2_ humidified atmosphere.

Primary hepatocyte cultures were obtained from male Wistar rats (body weight, 220 ± 20 g, 3 months of age, National Breeding Center, Sofia, Bulgaria) sacrificed under sodium pentobarbital anesthesia (60 mg/kg, i.p.). *In situ* liver perfusion and cell isolation were performed as previously described (Kondeva-Burdina et al., 2014). All procedures were approved by the Bulgarian Food Safety Agency (No. 304 valid until June 28, 2026) and by the Animal Ethics Committee of the University of Sofia (No. 220, April 13, 2021). The principles stated in the European Union Guidelines for the Care and the Use of Laboratory Animals, Directive 2010/63/EU were strictly followed throughout the experiment.

HepG2 and rat hepatocytes viability after 24, 48, or 72 h treatment with Dox and Sdox were investigated by using the 3-(4,5-dimethylthiazol-2-yl)-2,5-diphenyltetrazolium bromide (MTT) assay (Mosmann, 1983) and the trypan blue exclusion assay (Strober, 2001), respectively.

The extracellular release of lactate dehydrogenase (LDH), considered an index of cell damage and necrosis, was measured in both cell models as previously reported (Decker and Lohmann-Matthes, 1988; Fau et al., 1992). Based on its extent, the magnitude of the cytotoxic effect was classified as follows: weak cytotoxicity (20–40% decrease in viability), medium cytotoxicity (40–60% decrease in viability), and strong cytotoxicity (60–80% decrease in viability) (López-García et al., 2014).

Gluthatione (GSH) depletion was evaluated after 24, 48, or 72 h treatment with both anthracyclines. Isolated rat hepatocytes were centrifuged at 400 *g* for 3 min, and the pellet was used for measuring intracellular GSH by means of the spectrophotometric method based on the conversion of the sulfhydryl reagent 5,5′-dithiobis-(2-nitrobenzoic acid) (DTNB) to the yellow derivative 5′-thio-2-nitrobenzoic acid (TNB) at 412 nm (Fau et al., 1992).

Lipid peroxidation was estimated by evaluating malondialdehyde (MDA) in the culture supernatant treated with 0.67% (w/v) 2-thiobarbituric acid (TBA). The absorbance was measured at 535 nm, and the amount of TBA reactants was calculated using a molar extinction coefficient of MDA 1.56 × 10^5^ M^−1^ cm^−1^ (Fau et al., 1992).

### *In Vitro* hERG Study

hERG-HEK293 cells (BPS Bioscience, San Diego, United States), expressing hERG K^+^ channel (Kv11.1), were cultured in Minimal Essential Medium (MEM) supplemented with 10% fetal bovine serum, 1% non-essential amino acids, 1% Na pyruvate, 1% penicillin/streptomycin, and 400 μg/ml geneticin (Lazzerini et al., 2021).

The bath solution contained (in mM): 136 NaCl, 5.4 KCl, 10 HEPES, 10 D-glucose ∙ H_2_O, 1 CaCl_2_ ∙ 2 H_2_O, 1 MgCl_2_ ∙ 6 H_2_O, pH 7.4 with NaOH, whereas the pipette solution consisted of (in mM): 130 KCl, 10 EGTA, 10 HEPES, 5 MgATP, 1 MgCl_2_ ∙ 6 H_2_O, pH 7.2 with KOH.

Whole-cell recording was performed as previously described (Lazzerini et al., 2021), and a depolarizing step from a holding potential of −80–20 mV was applied to elicit the Kv11.1 current. Tail current was evoked by repolarizing the cell membrane to −50 mV for 4 s.

Kv11.1 current values were corrected for leakage using 1 µM E4031, which completely blocked hERG channels.

### Zebrafish Embryo Toxicity Studies

Adult wild-type Tübingen zebrafish (*Danio rerio*) were used in all experiments and maintained at 28 ± 1°C under continuous water aeration and filtering and artificial light with a 12/12 h dark/light cycle, according to The Zebrafish Book (Westerfield, 2000). The fish were regularly fed twice a day with commercial dry-flake food (TetraMin™ flakes; Tetra Melle, Germany) supplemented with *Artemia nauplii* once a day. The day before spawning, males and females were placed in separated breeding tank at a ratio of 1:2 before the onset of darkness and left undisturbed overnight. At the onset of light, the separators were removed from the breeding tanks. After 30 min, the eggs were collected, rinsed twice from debris using fresh embryo medium (Instant ocean), and transferred into Petri dishes containing the embryo medium.

Fertilized eggs were selected under a binocular stereomicroscope (PXS-VI, Optica) and transferred into 24-well plates. Twelve embryos were placed in each well in a 2-ml of embryo medium, treated with either Dox or Sdox at 6 h post fertilization (hpf). The embryo medium and 0.1% DMSO were used as controls. Treated embryos and controls were then incubated at 28 ± 0.5°C. Each experiment was repeated three times from three independent breedings. In each experiment, at least 3 wells with 12 embryos per treatment per concentration were used.

The zebrafish embryo toxicity assay was performed when fertilization rate was ≥90%. An assay was considered valid if the overall survival of embryos in negative controls was ≥90% until hatching.

All embryos were inspected for morphological characteristics at different developmental stages (48 and 72 hpf) as described by Kimmel (1989). Lethal and teratogenic effects were recorded according to the Organisation for Economic Co-operation and Development (OECD) (236) guidelines for testing of chemicals. Mortality was determined by counting embryos with or without a visible heart beat at 72 hpf. For visual documentation of the malformations and heartbeat, embryos were imaged and recorded using a camera connected to the microscope (CKX41; Olympus, Tokyo, Japan). Each heartbeat was manually counted for 20 s and multiplied by 3 to calculate heart rate (beat/minute) (Hoage et al., 2012). At least three embryos per group were recorded and their heart beat counted. Teratogenic effects were recorded if the fingerprint end-point was observed in at least 50% of all embryos showing teratogenic effects and if these effects were concentration-related.

Zebrafish experiments were conducted under the standard national and EU regulations.

### Materials

The materials used included DMEM, RPMI 1640, FBS, L-glutamine, penicillin, streptomycin, Dox, sodium pyruvate, geneticin, accutase, E-4031, trypsin/EDTA, MTT, SRB, Tris, and verapamil (Sigma-Aldrich, Milan, Italy; Sigma Aldrich, Germany), MEM, non-essential amino acids, FBS (Gibco, Life Technologies, New York, United States), amd LDH cytotoxicity Kit (TaKaRa, Germany).

Sdox (2-((2S,4S)-4-(((2R,4S,5S,6S)-4-amino-5-hydroxy-6-methyltetrahydro-2H-pyran-2-yl)oxy)-2,5,12-trihydroxy-7-methoxy-6,11-dioxo-1,2,3,4,6,11-hexahydrotetracen-2-yl)-2-oxoethyl 4-(4-phenyl-3-thioxo-3H-1,2-dithiol-5-yl)benzoate) was synthetized as previously reported (Chegaev et al., 2016).

### Statistical Analyses

Statistical analysis of data was performed with GraphPad Prism 6 Software (GraphPad Software Inc., San Diego, CA, United States). Comparisons among groups were performed by Student’s *t*-test for unpaired samples, one-way ANOVA with Dunnett’s multiple comparisons *post hoc* test, or multiple *t*-tests with a Holm–Sidak correction, assuming similar dispersions. Values of *p* < 0.05 were considered statistically significant.

## Results

### *In Silico* Studies

**FIGURE 1:**
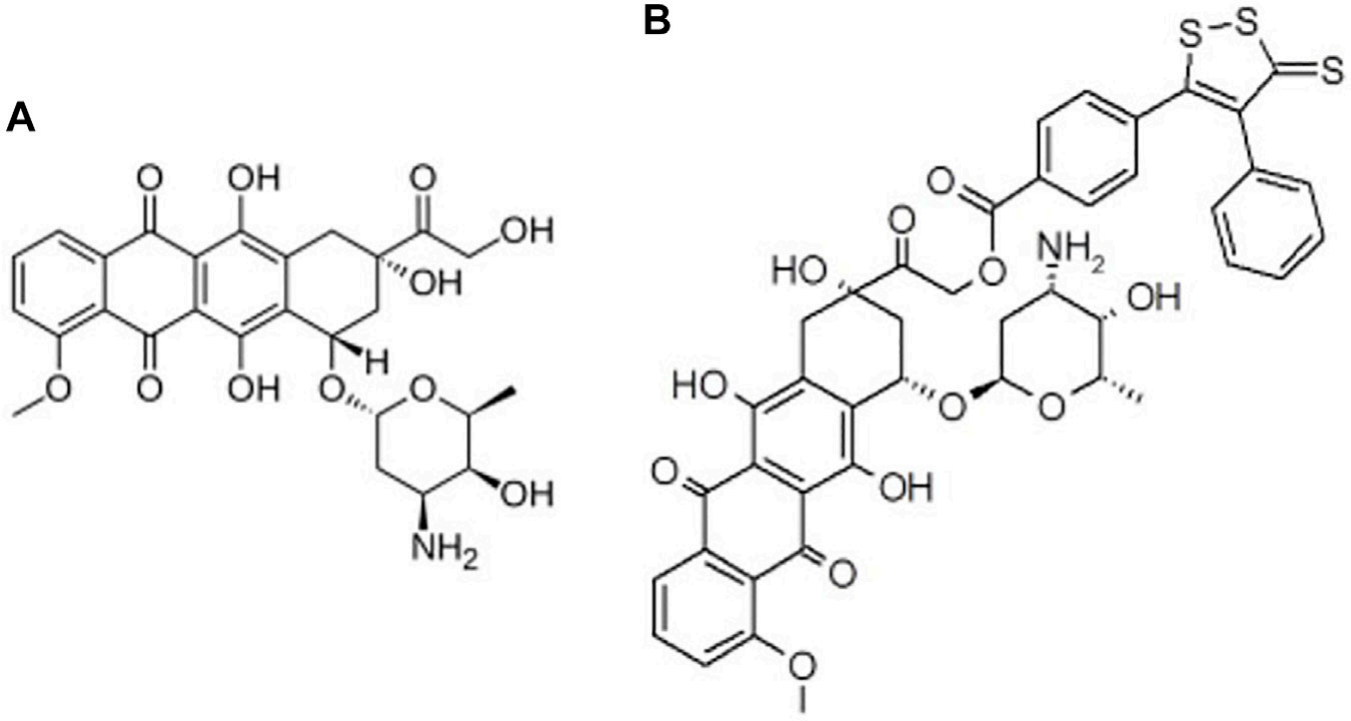
2D structures of **(A)** Dox and **(B)** Sdox.

The *in silico* studies aimed at a preliminary evaluation of the ADME/Tox properties of Sdox to guide further experimental studies. A multistep procedure was used involving the following types of drug profiling: physicochemical and ADME; drug safety; metabolic transformations; docking and binding to PXR and SULT; and off-targets prediction. In all steps, the results obtained for Sdox (Figure 1) were compared to those calculated for Dox to help in assessing the reliability of predictions.

### Physicochemical Characterization and ADME

Table 1 summarizes the results on the physicochemical characterization of the studied compounds by ACD/Percepta.

**TABLE 1:** Physicochemical profiles of Dox and Sdox as predicted by ACD/Percepta software (differences are shaded).

Physicochemical parameter	Dox		Sdox	
	Value	Category/range	Value	Category/range
logP^a^	0.92	Optimal (−1< ÷ ≤ 4.2)	6.32	Very lipophilic (>5)
MW	543.52	Bad (>500)	855.95	Bad (>500)
No. of H-bond donors	7	Bad (>5)	6	Bad (>5)
No. of H-bond acceptors	12	Bad (>10)	13	Bad (>10)
No. of rotational bonds	5	Good (≤10)	10	Good (≤10)
No. of rings	5	Bad (>4)	8	Bad (>4)
Lipinsky rule of 5 (No. of violations)	3	Bad (less than 2 rules apply)	4	Bad (less than 2 rules apply)
Lead-like rule^b^ No. of violations)	3	Bad (less than 2 rules apply)	4	Bad (less than 2 rules apply)
Solubility, mg/ml	12.7	Soluble	5.10^–5^	Highly insoluble

a Consensus algorithm combining classical fragment- and similarity-based approach.

b Applies to the following thresholds: −2 ≤ logP <4.2; MW < 460; No. of H-bond donors ≤5; No. of H-bond acceptors ≤9. The main differences are shaded.

The results point to two main differences in the physicochemical properties between Dox and Sdox: lipophilicity and solubility (shaded rows in Table 1). Considering the importance of the partition coefficient logP, this fundamental molecular descriptor was calculated by several software programs employing different predictive algorithms (Table 2). The calculated logP values of Dox differed depending on the applied algorithms; however, the average value of 1.13 obtained by the different methods was close to the experimental one, corresponding to 1.27 (Viswanadhan et al., 1989), suggesting reliable prediction of this descriptor. In addition, to consider the property of the compound at physiological pH, the distribution coefficient logD was calculated (Table 2). Obviously, the presence of the H_2_S-donor group has an essential impact on the lipophilicity of Sdox, the compound remaining highly hydrophobic even at pH = 7.0.

**TABLE 2:** Calculated logP and logD (at pH = 7.0) of Dox and Sdox using different software.

Software	logP/logD	
	Dox	Sdox
ACD/Percepta^a^	0.92/−0.93	6.05/4.20
MOE	0.96/−0.26	6.79/5.57
Marvin	1.50/0.10	7.14/5.73
Average value	1.13/−0.32	6.75/5.30
Experimental value^b^	1.27/n.d.^c^	n.d.

a Consensus algorithm combining classical fragment-based and similarity-based approach.

b (Viswanadhan et al., 1989).

c Not determined.

The results of ADME profiling showed that both drugs possess poor permeability across Caco-2 monolayers (Pe ≤ 1.10–6 cm/s at pH = 7 and 500 rpm stirring rate) and their capacity for BBB penetration (the model takes into account only the BBB governed by passive diffusion) scored a low value, suggesting inactivity in the CNS.

While Dox was ranked as strongly bound to plasma proteins (80% < PPB ≤ 90%), Sdox was estimated as “undefined” with RI < 0.3. It should be noted that the predictions related to HIA were not considered, taking into account that Dox (and presumably Sdox) is not administrated orally.

### Drug Safety

The predictions of the ability of Sdox to interact with the membrane transporter P-gp and the major CYP450 isoforms responsible for drug metabolism (CYP1A2, 2C9, 2C19, 2D6, 3A4) were classified Dox as a P-gp substrate and as a non-inhibitor of all CYP450 isoforms. For Sdox, all predictions fell into the category “undefined” with the exception of CYP1A2 and CYP2D6 for which the compound was estimated as a non-inhibitor.

Cardiac and hepatic drug safety profiles were further predicted using ACD/Percepta and Derek Nexus expert systems (Table 3). The reported values showed that, according to ACD/Percepta estimation, both compounds had low probability to bind to hERG channel; however, these predictions were outside the applicability do-main of the model used for prediction. The results of Derek Nexus gave plausible levels for Sdox cardiotoxicity and hepatotoxicity and a higher likelihood level for Dox hepatotoxicity (probable).

**TABLE 3:** Toxicity predictions of Dox and Sdox.

Toxicity	Dox	Sdox
ACD/Percepta: 0.33 < Score ≤0.67 (undefined)		
hERG inhibition (K_i_ < 10 μM, patch-clamp method)	Score = 0.36 *p* = 0.01 RI = 0.28	Score = 0.43 *p* = 0.05 RI = 0.15
Derek Nexus: likelihood levels		
Cardiotoxicity in mammals	plausible	plausible
Hepatotoxicity in mammals	probable	plausible

For Score, p, RI, and likelihood levels, see *Materials and Methods*.

### Metabolic Transformations

The prediction of the biotransformations resulted in 37 different metabolites of Dox and 69 of Sdox. Supplementary Tables S1, S2 represent the metabolic trees, and Supplementary Tables S3, S4 illustrate the structures of predicted metabolites. Duplicated metabolites appeared more than once at different steps of the biotransformation cascade. Reproduction of the original input structures during the liver enzymatic reactions was also predicted. The comparative analysis of Phase I metabolic profiles of Dox and Sdox underlined 19 shared metabolites (Supplementary Table S5) related to their common molecular scaffold.

By focusing on the “on path likelihood” of the predicted biotransformations, all 37 Dox and 54 Sdox metabolites were outlined as plausible. Probable likelihood of occurrence was assigned to the remaining 15 Sdox metabolites. Among them, seven were shared in the Dox metabolic tree. Doxorubicinol, one known Dox metabolite, was found in the Meteor output for Dox, and a probable Sdox metabolite, thus supporting the predictions made.

**FIGURE 2:**
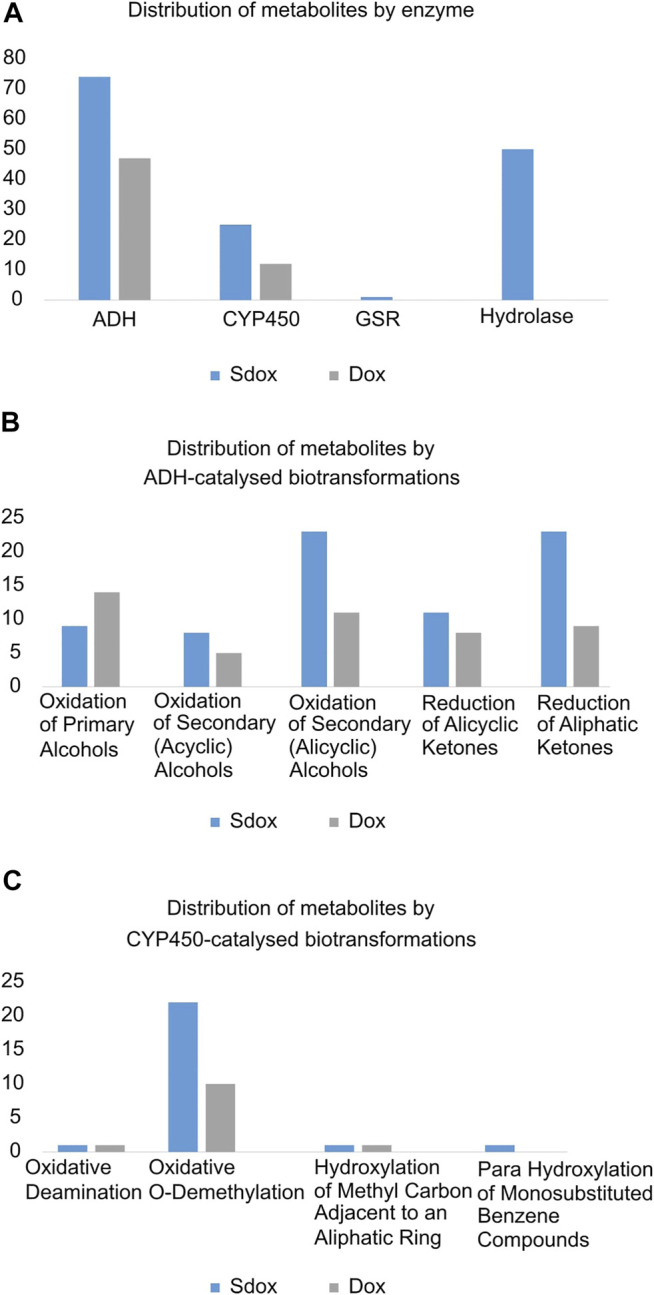
Comparative analysis of Phase I biotransformations of Sdox and Dox. Distribution of metabolites by **(A)** enzyme, **(B)** ADH-catalysed, **(C)** CYP450-catalysed biotransformations.

A comparative analysis of the individual biotransformations frequency was performed for both dox and Sdox (Figure 2). Eleven different biotransformations were predicted to be catalysed by four enzymes. Nearly 80% (47) of Dox metabolites resulted from reactions catalyzed by the alcohol dehydrogenase (ADH), and the remaining 20% (12) resulted from CYP450-catalysed biotransformations. The Sdox metabolic profile revealed 49% (74) of the reactions involving ADH, 33% (50) of the reactions catalyzed by hydrolase, 17% (25) by CYP450, and only one reaction mediated by the glutathione-disulfide reductase (GSR) (Figure 2A). Overall, the ADH-catalyzed oxidation of secondary (acyclic) alcohols and the reduction of aliphatic ketones accounted for twice more metabolites in the Sdox profile than in the metabolic tree of Dox (Figure 2B). A similar result was obtained with the oxidative O-demethylation operated by the CYP450 system (Figure 2C).

**FIGURE 3:**
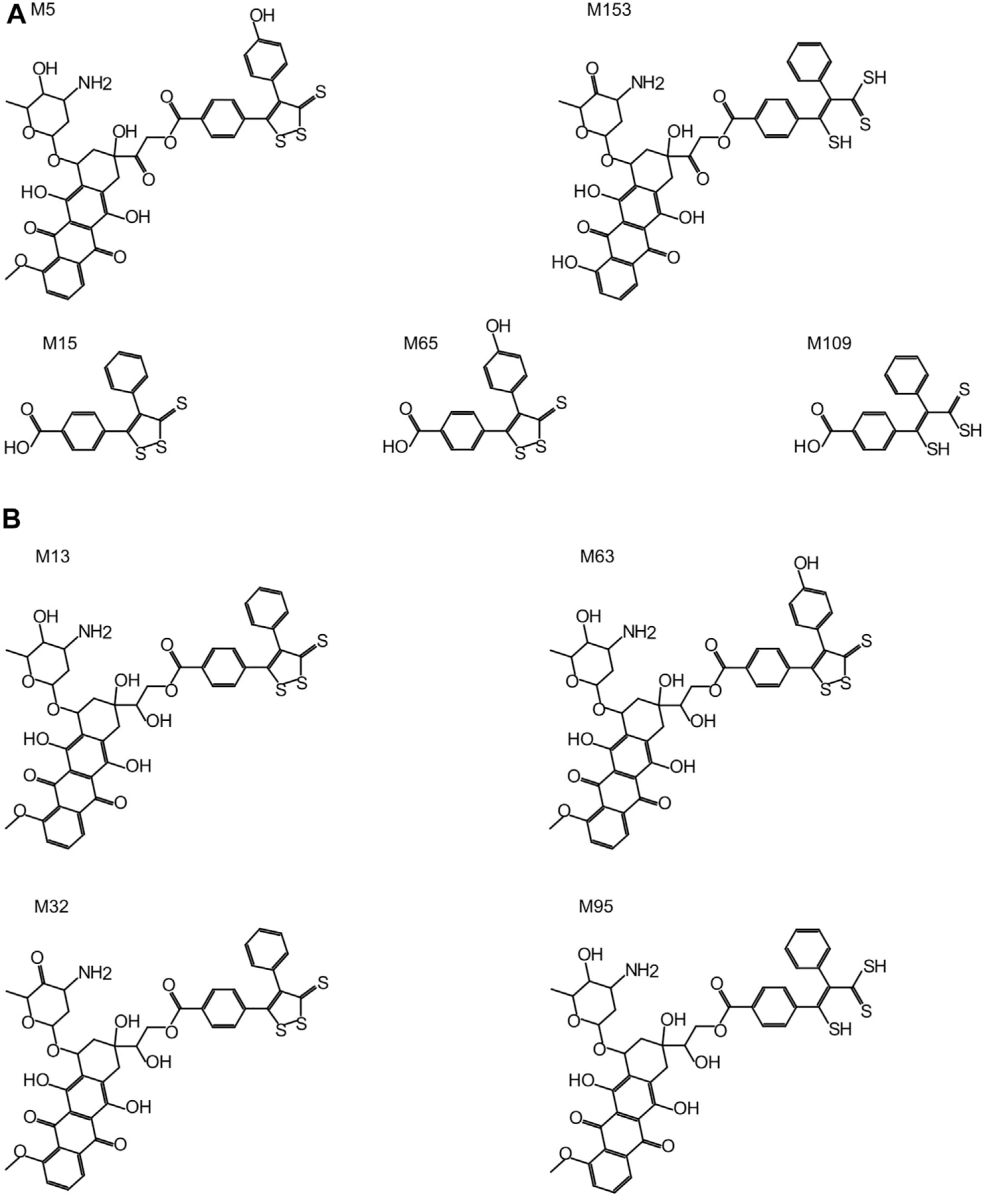
Sdox metabolites (M). **(A)** Characteristic Sdox metabolites possibly acting as H_2_S donors. **(B)** Plausible Sdox metabolites with scaffolds identical or very similar to doxorubicinol.

All Sdox biotransformations involving hydrolases were related to “hydrolysis of acyclic carboxylic esters” and were responsible for the enzymatic cleavage of the 3-thioxo-3H-1,2-dithiol-5-yl group (or its metabolites) from the Dox scaffold (in its original or metabolized form). These reactions were listed exclusively in the Sdox profile. They were predicted to result in two plausible (M65 and M109) and one probable (M15) metabolites, possibly relevant to the hydrogen sulfide release from Sdox. Therefore, these metabolites were suggested to act as H_2_S donor substructures (Figure 3A). Several Sdox metabolites either reproduced the scaffold of the known and most relevant Dox metabolite doxorubicinol or included its derivative presenting a carbonyl instead of a carboxyl substituent on the glycosidic moiety (Figure 3A, M13 and M32). Other contained a metabolized form of the H_2_S donor group attached to a core sub-structure, reproducing doxorubicinol (Figure 3B, M63 and M95).

The above predictions prompted further studies to evaluate Sdox hepatotoxicity, hERG liability, and CYP450 inhibition.

### Docking and Binding to Pregnane-X-Receptor and Sulfotransferase

Docking and binding of both anthracyclines (Supplementary Figure S1) and some of their predicted metabolites to PXR and SULT were performed.

Glide (XP) was not able to calculate docking scores for Sdox and some of its metabolites to PXR and SULT, possibly due to inability of the applied algorithm to fit them properly into the respective binding sites because of their large molecular size (Table 4).

**TABLE 4:** Docking scores of Dox and Sdox binding to pregnane-X-receptor (PXR, PDB ID:1m13) and sulphotransferase (SULT, PDB ID: 2a3r) (kcal/mol).

Compound	PXR			SULT		
	Glide XP	Vina	AutoDock	Glide XP	Vina	AutoDock
Threshold	**−7.7**	**-10.3**	**-12.5**	**-6.3**	**-6.0**	**-7.5**
Dox	−11.89	−8.8	−8.46	-	−3.2	−8.83
Sdox	-	−8.5	−9.28	-	+0.7	−2.05
M5	-	−6.8	−9.77	-	+7.3	+0.19
M13	-	−6.9	−9.35	-	+5.3	−2.20
M15	−8.39	−7.6	−8.0	−6.21	−7.7	−7.51
M32	-	−7.8	−9.58	-	+3.7	−2.54
M63	-	−6.7	−8.89	-	+5.0	+1.78
M65	−8.15	−7.2	−7.43	−6.74	−8.2	−8.11
M95	-	−7.3	−9.33	-	+4.8	−0.98
M109	−6.66	−6.7	−7.57	−6.33	−7.2	−7.31
M153	-	−8.2	−9.81	-	+4.4	−2.13

Docking score of Dox, Sdox, and Sdox common metabolites (M) calculated according to three different software (Glide XP, Vina, and AutoDock). Values in bold indicate thresholds.

According to Vina and AutoDock, Dox, Sdox, and most of the Sdox metabolites displayed comparable docking scores for PXR binding, although lower than the previously determined threshold. Instead, Dox (only with AutoDock) and Sdox metabolites M15, M65, and M109 showed better scores for SULT binding compared to Sdox.

### Off-Target Prediction

Dox forms complexes with DNA by intercalation between base pairs, and it inhibits TOPO II activity by stabilizing the DNA-topoisomerase II complex, while Sdox releases H_2_S in the ER. Therefore, any other interaction with proteins must be considered an off-target effect (side effect, non-therapeutic, toxicological, etc.) unless a proof of the pharmacological (therapeutic) effect is reported.

Predictive *in silico* off-target profiling for Sdox was performed by using the reverse docking method, to assess possible, relevant differences between Dox and Sdox towards several cancer targets. Neither a standard protocol nor a list of recommended targets to study is reported in the literature. Based on our prior findings (Silveira-Dorta et al., 2015), we selected for docking purposes our in-house set of 85 common human cancer targets, which are available in PDB (Supplementary Table S6), whose resolution is valid for docking studies. Although not covering all possible protein–drug interactions, the present approach showed how small chemical differences between Dox and Sdox led to relevant changes in the selectivity towards cancer targets.

Among the top 10 ranked interactions (Table 5), only PKC-α was common to both compounds.

**TABLE 5:** Top-ranked drug-protein interactions obtained for Sdox and Dox.

	Sdox			Dox		
Entry	DS (kcal/mol)	PDB ID	Protein	DS (kcal/mol)	PDB ID	Protein
1	−11,344	2euf	CDK6	−15,434	4o2b	β-tubulin (VBS)
2	−10,017	2jed	PKC-ζ	−11,537	1o6k	AKT2
3	−9,843	1y6a	VEGFR2	−9,764	2b7a	JAK2
4	−9,667	2xrw	MAPK 8	−9,445	2rcw	PARP 1
5	−9,500	3wpn	KIF11	−9,149	3gp0	MAPK 11
6	−9,358	1rr8	DNA Topo I	−8,849	3iw4	PKC-α
7	−9,293	2etk	ROCK1	−8,769	1boz	DHFR
8	−9,132	1ua2	CDK7	−8,751	1uym	HSP 90-β
9	−9,115	2ivu	RET	−8,747	1cm8	MAPK 12
10	−8,831	3iw4	PKC-α	−8,725	1njs	GART

**FIGURE 4:**
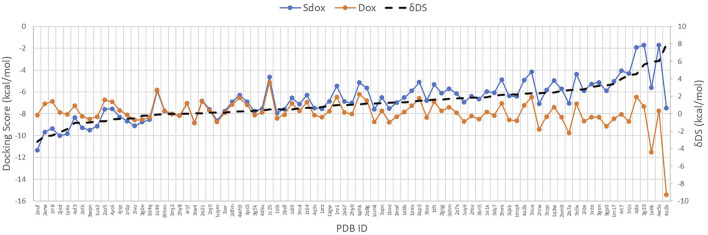
Docking score of Sdox and Dox against 85 representative cancer targets.

Figure 4 shows the DS for both drugs against the selected targets, plotted in order of increasing ΔDS with the largest ΔDS differences being summarized in Table 6. The results predicted a significant difference (>2 kcal/mol) for the binding interaction with CDK6, MAPK 8, DNA Topo I, and PKC-ζ. A higher ΔDS (>5 kcal/mol) was found for *β*-tubulin (VBS), tankyrase (TNKS), GSK-3 β, and Cyclin D3 (CCND3). Thus, the proteins CDK6, MAPK 8, DNA Topo I, and PKC-ζ were predicted to represent preferential targets for Sdox, whereas Dox was anticipated to bind preferentially the proteins *β*-tubulin (VBS), TNKS, GSK-3 β, and CCND3.

**TABLE 6:** Top-ranked ΔDS values obtained for Sdox and Dox.

Preferred drug	PDB ID	DS (kcal/mol)		ΔDS	Protein
		Sdox	Dox	(kcal/mol)	
Sdox	2euf	−11,344	−8,119	−3,225	CDK6
	2xrw	−9,667	−7,096	−2,571	MAPK 8
	1rr8	−9,358	−6,880	−2,478	DNA Topo I
	2jed	−10,017	−7,897	−2,120	PKC-ζ
Dox	4o2b	−7,477	−15,434	7,957	β-tubulin (VBS)
	4w5s	−1,692	−7,729	6,037	TNKS
	1o6k	−5,610	−11,537	5,927	GSK-3 beta
	3g33	−1,705	−7,340	5,635	CCND3

On the left side of the graph, we can observe the targets for which hypothetically Sdox binds stronger than Dox. The targets with more affinity for Dox than for Sdox are reported on the right-hand side.

### *In Vitro* Studies

To further characterize the differential impact in terms of efficacy, metabolism, and safety between Dox and Sdox, four parameters were analyzed *in vitro*: the efficacy in cell lines overexpressing P-gp, the inhibition of CYP3A4, involvement in Dox and several drugs metabolism, and the toxic effects on hepatocytes and that on hERG channel.

### P-Glycoprotein Assay

The effect of P-gp overexpression in the antiproliferative activity of Sdox is shown in Table 7. GI_50_ values recorded in P-gp overexpressing and wild-type cell lines and their ratio (resistance factor, Rf) are given. In the absence of verapamil, Sdox was slightly affected by P-gp overexpression (Rf = 3.5) as compared to Dox and, in particular, to paclitaxel (Rf = 564). Co-treatment with verapamil caused a decrease in Rf for all anti-mitotic drugs. This result is consistent with the data on Table 5.

**TABLE 7:** Antiproliferative activity (GI_50_) of Sdox, Dox, and tubulin-interacting drugs in SW1573 and SW1573/P-gp cell lines.

Drug	w/o verapamil			w Verapamil		
	*SW1573*	*SW1573/P-gp*		*SW1573*	*SW1573/P-gp*	
	GI_50_ (nM)		*Rf*	GI_50_ (nM)		*Rf*
Sdox	873 ± 410	3,015 ± 531	3.5	975 ± 330	1757 ± 616	1.8
Dox	70 ± 8.6	1,601 ± 169	23	26 ± 8.2	185 ± 23	7.2
Paclitaxel	0.53 ± 0.22	298 ± 113	564	0.46 ± 0.21	0.31 ± 0.15	0.7
Colchicine	67 ± 15	2,366 ± 937	35	29 ± 12	154 ± 39	5.2
Vincristine	3.9 ± 1.5	86 ± 15	22	0.60 ± 0.11	1.52 ± 0.18	2.5
Vinblastine	0.94 ± 0.35	16 ± 4.7	17	0.55 ± 0.11	1.02 ± 0.45	1.9

Rf = GI_50_(SW1573/P-gp)/GI_50_(SW1573), data are expressed as mean ± SD (*n* = 3–5).

### *In Vitro* Inhibition of Human CYP3A4

Previous studies have suggested that CYP3A4 enzyme plays a role in the oxidative metabolism of Dox in humans (Kivistö et al., 1995).

All compounds exhibited CYP3A4 inhibition potency comparable to ketoconazole [IC_50_ 6.8 µM, 95% confidence interval (CI) 3.5–10.1 for Dox; 3.1 µM, 95%CI 1.9–4.3 for Sdox; 13.7 µM, 95%CI 3.8–23.6 for ketokonazole] with 95% CI overlapping for all compounds (*p* > 0.05). Thus, Dox and Sdox inhibit CYP3A4 to a degree comparable to the reference inhibitor ketoconazole.

### *In Vitro* Safety Evaluation of Sdox and Dox on HepG2 Cells and Primary Rat Hepatocytes

To characterize deeper the safety profile of Sdox vs. Dox, a comparative *in vitro* evaluation of both compounds on two complementary liver-derived cell models, the HepG2 cells and the freshly isolated rat hepatocytes, was performed. HepG2 cells are non-tumorigenic cells with high proliferation rates and an epithelial-like morphology that perform many differentiated hepatic functions (Donato et al., 2015).

The effects of Dox and Sdox on HepG2 cell viability were assessed by using the MTT assay (Table 8). At all scheduled times, the estimated IC_50_ values demonstrated that HepG2 cells were more sensitive to Dox than to Sdox (Table 8).

**TABLE 8:** Effects of Dox and Sdox on HepG2 cell viability.

Compound	IC_50_ (μM)		
	24 h	48 h	72 h
Dox	6.11 ± 0.66	0.46 ± 0.03	0.19 ± 0.01
Sdox	8.69 ± 0.31**	3.21 ± 0.16***	1.39 ± 0.03***

Potency of compounds is expressed as estimated IC_50_ values (µM). Data are reported as mean ± SD, of 3 independent experiments. **p < 0.01, ***p < 0.001 vs. Dox (Student’s *t*-test for unpaired samples).

**FIGURE 5:**
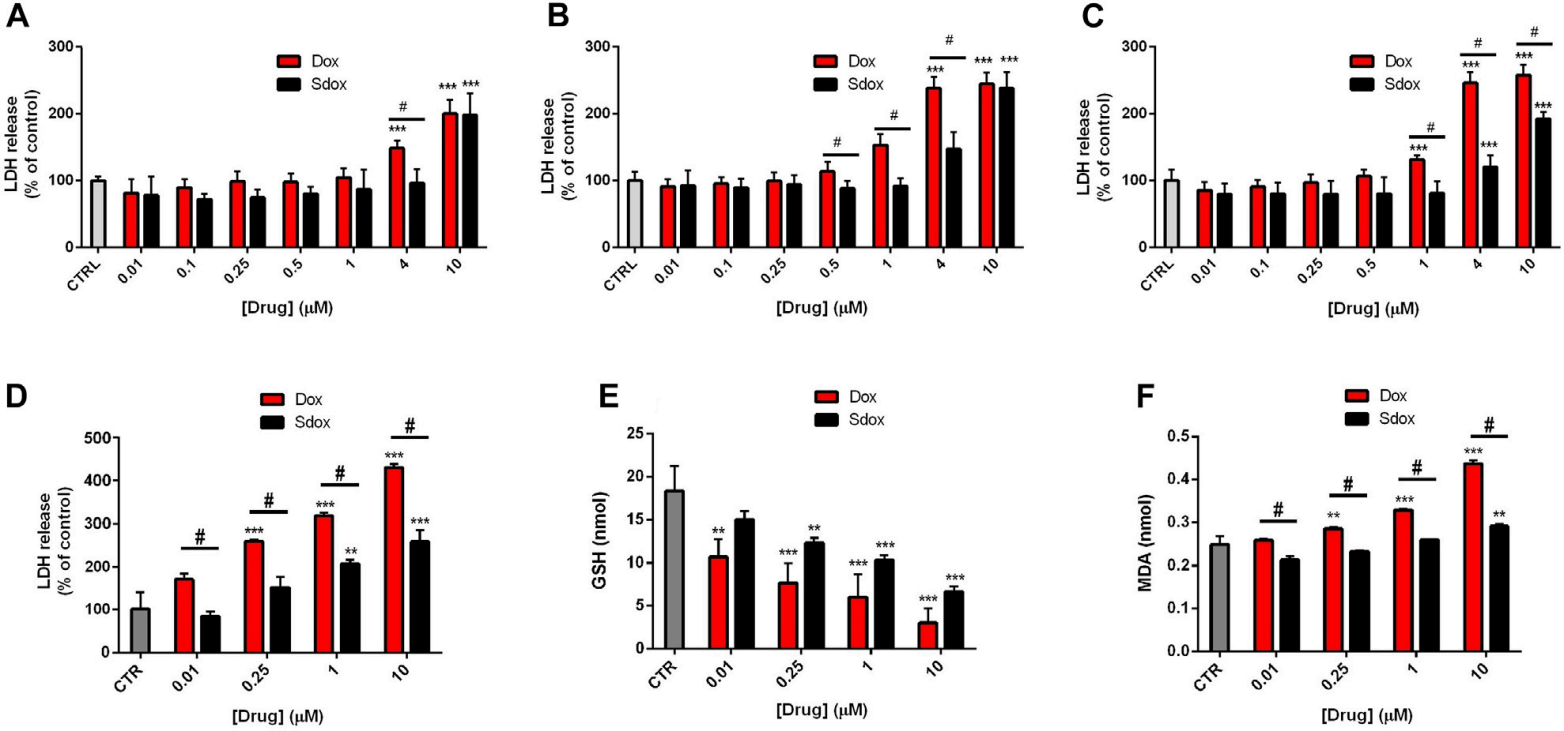
Effects of Sdox and Dox treatment on HepG2 cells and primary rat hepatocytes. LDH release after **(A)** 24 h, **(B)** 48 h, and **(C)** 72 h treatment with Dox and Sdox in HepG2 cells. **(D)** LDH release and oxidative stress estimated by measuring **(E)** reduced GSH and **(F)** MDA in primary rat hepatocytes. Data are means ± SD of triplicate assays (*n* = 3). *p < 0.05; **p < 0.01; ***p < 0.001 vs. controls, one-way ANOVA with Dunnett’s post-test. #*p* < 0.05 vs. Dox, multiple *t*-test, corrected by the Holm–Sidak method.

LDH release from cultured HepG2 cells, an indication of cell membrane injury, was examined. After 24 and 48 h of treatment, Sdox increased LDH release only at the maximum concentration tested (10 μM) and at 72 h also at 4 μM. However, Dox became cytotoxic at concentrations lower than those of Sdox, regardless of timing of exposure (Figures 5A–C).

To further confirm the data obtained in HepG2 cells, we analyzed more in depth a second non-transformed liver model, i.e., primary isolated rat hepatocytes, which, although characterized by short life-span, exhibit higher levels of expression and activity of drug-metabolizing enzymes than HepG2 cells (Soldatow et al., 2013; Polidoro et al., 2021).

Dox and Sdox reduced the number of viable cells, estimated by the trypan blue exclusion assay, with IC_50_ values of 9.68 ± 0.89 µM and 16.49 ± 1.01 µM, respectively (*p < 0.05 vs. Dox, Student’s t-test for unpaired samples) and increased the release of LDH in a concentration-dependent manner, Dox being significantly more potent and toxic than Sdox (Figure 5D). Moreover, Sdox produced a lower decrease in the reduced GSH and a lower increase in the MDA levels, considered an index of lipid peroxidation (Figures 5E,F), indicating that it causes a lower oxidative stress as compared to Dox.

The data from *in vitro* hepatic safety evaluation on both HepG2 cells and isolated hepatocytes are consistent with the *in silico* predicted drug safety profiles of Sdox vs. Dox in terms of hepatic drug safety (shown in Table 3).

### Effect of Dox and Sdox on hERG (KV11.1) Current

**FIGURE 6:**
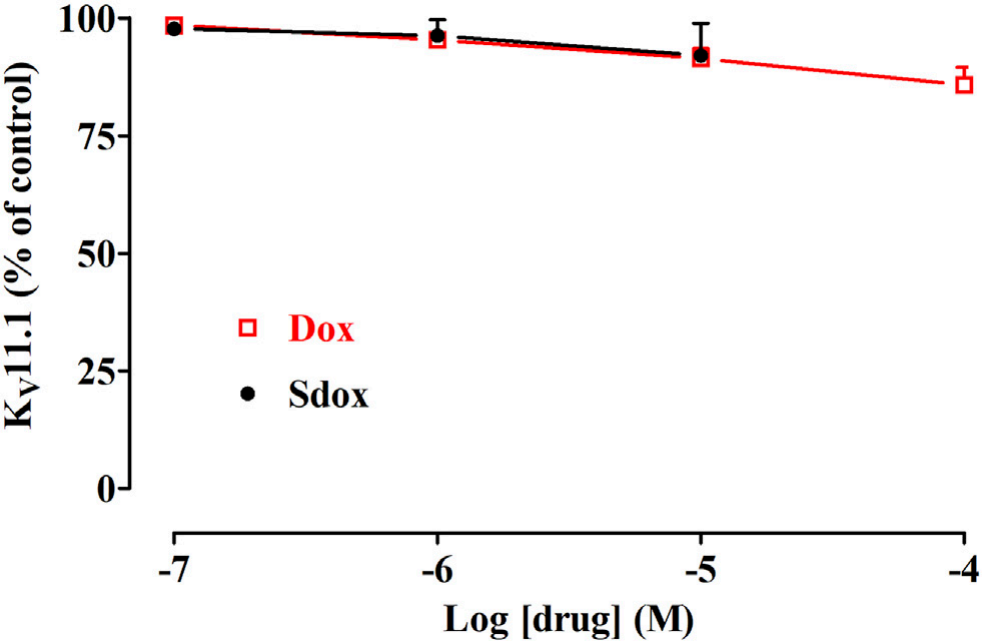
Effect of Dox and Sdox on K_V_11.1 (hERG) current recorded in hERG-HEK293 cells. Concentration-dependent effect of Dox and Sdox of hERG tail currents. On the ordinate scale, current amplitude is reported as a percentage of the value recorded just before the addition of the first concentration of the drug. Data points are the mean ± SD (*n* = 6).

Dox (up to 100 μM) and Sdox (up to 10 μM) did not affect hERG currents recorded in hERG-HEK293 recombinant cells by using the patch-clamp technique (Figure 6).

### Zebrafish Embryo Toxicity Studies

**FIGURE 7:**
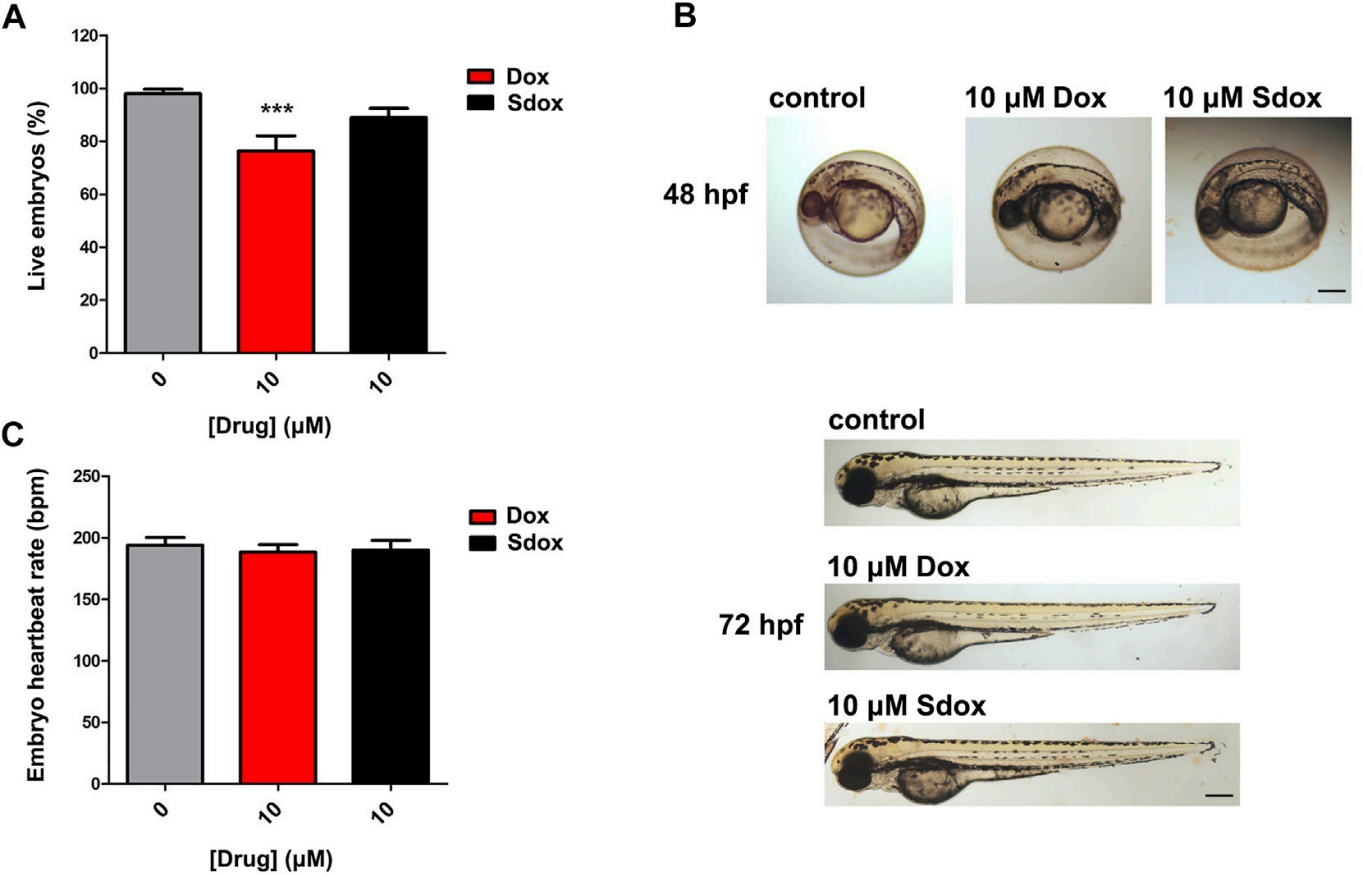
Effect of Dox and Sdox on zebrafish. **(A)** Seventy-two hours post fertilization zebrafish embryos survival. **(B)** Representative images of Dox- or Sdox-treated zebrafish embryos at 48 and 72 hpf. Images of zebrafish embryos at 72 hpf are panorama images. Scale bar = 500 µm. **(C)** Heartbeat rate in 72 hpf zebrafish embryos after treatment with Dox and Sdox. Columns are mean ± SD. ***p < 0.001 vs. control (0), one-way ANOVA with Dunnett’s post-test.

To further deepen the comparison between the toxicity of Sdox and Dox, the lethality induced by the drugs, applied at 6 hpf, was monitored in zebrafish embryos at 72 hpf (Figure 7). As shown in Figure 7A, 10 µM Dox markedly reduced the number of live embryos, whereas Sdox was ineffective with nearly 90% of embryos alive. Similar results were observed at 96 hps (data not shown).

The toxic effects of both anthracyclines, at 10 µM concentration, on embryo development were analyzed at 48 and 72 hpf. In particular, at 48 hpf, neither visible teratogenic effects nor differences in hatching rate were observed (Figure 7B), and at 72 hpf, Dox and Sdox did not display developmental toxicity in zebrafish embryos (Figure 7B). Consistently, neither compound affected the embryo heartbeat rate at 72 hpf when applied at 10 µM concentration (Figure 7C).

As expected, when higher concentrations were applied (up to 100 µM), a Dox-induced concentration-dependent developmental toxicity was evident. Notably, cardiotoxicity was manifested through the presence of pericardial edema and deformity of the heart, and a reduction of zebrafish embryo heartbeat rate (data not shown). Instead, Sdox was tested up to 10 μM because a precipitate was observed at higher concentrations.

To test whether the presence of chorion can interfere with the diffusion of Sdox and Dox, zebrafish embryos were manually dechorionated at 24 and 48 hpf and exposed to either drug. Malformations detected in dechorionated embryos were similar to those observed in embryos with intact chorion.

## Discussion

Sdox and its liposomal formulation were recently proposed as an innovative and effective therapeutic tools against P-gp-overexpressing/Dox-resistant tumors (Bigagli et al., 2018; Gazzano et al., 2019).

The present study evaluated Sdox drug-like characteristics and ADME/Tox properties *in silico*, *in vitro*, and *in vivo*.

The *in silico* physicochemical profiling of Sdox demonstrates that it maintains some of Dox properties but has specific features different from its parental drug that may constitute an advantage in terms of reduced toxicity, increased efficacy against P-gp-overexpressing cells, metabolism, and reduced off-target effects.

For instance, Sdox possesses high lipophilicity and low solubility compared to Dox, suggesting a different pharmacokinetic behavior. The lipophilicity of anthracyclines has been previously investigated using spectrofluorimetric method to evaluate cell uptake, and a positive correlation between their influx rates and logP has been reported (Rivory et al., 1996). Thus, it may be expected that the higher lipophilicity of Sdox compared to Dox confers an increased uptake within tumor cells, also in the presence of high levels of P-gp, as demonstrated previously (Buondonno et al., 2019) and suggested by the higher cytotoxicity of Sdox compared to Dox in P-gp-overexpressing cells observed in the present work. On the other hand, the intracellular uptake of Dox from the solution was found to be higher than from liposomal formulation at therapeutically relevant concentrations (Kullenberg et al., 2021), while low solubility warns about the need of administering Sdox-like drugs as liposomal formulations to ensure a higher efficacy (Gazzano et al., 2019).

The *in silico* studies classified Dox as a non-inhibitor of all CYP450 isoforms included in prediction (CYP1A2, 2C9, 2C19, 2D6, and 3A4); Sdox was assigned as a non-inhibitor of two of them (CYP1A2 and 2D6). On the other hand, *in vitro* data showed that Dox and Sdox inhibited CYP3A4 comparably to ketoconazole, suggesting that both anthracyclines have relatively high affinity to the human CYP3A4 enzyme, isoform paramount to drug metabolism. This is an important information to acquire in the perspective of future clinical applications of Sdox-like drugs, since many oncological patients are treated with different drugs, and therefore, they are very susceptible to drug–drug interactions. To limit undesired drug–drug interactions, once again, the liposomal formulations of Sdox that have a higher tumor-to-healthy tissue delivery (Gazzano et al., 2019) are crucial.

Differences were also recorded in the effects of both drugs on the membrane transporters, including P-gp. Dox is a well-known P-gp substrate (Food and Drug Administration, 2013), while according to the *in silico* prediction, Sdox fell into the category “undefined” in relation to this protein.

In the present study, co-treatment of P-gp-overexpressing cells with the P-gp inhibitor verapamil demonstrated that Sdox is a substrate with low affinity for this transporter, and it is less affected (ca. 6.5 times) by P-gp when compared with its parent compound Dox. This agrees with previous studies demonstrating the efficacy of Sdox towards P-gp-overexpressing cells (Chegaev et al., 2016; Bigagli et al., 2018; Gazzano et al., 2019). It was proposed that Sdox may trigger the P-gp ubiquitination by altering the disulfide bonds in the protein structure and subsequently its conformation and stability (Buondonno et al., 2019; Gazzano et al., 2019). Indeed, results obtained in this study on Sdox metabolic transformations confirm the presence of metabolites that can cause P-gp sulfhydration.

Moreover, as PXR has a key role in the regulation of both drug metabolism and drug efflux by activating the expression of genes encoding CYP450 enzymes and drug efflux transporters (Ekins and Schuetz, 2002; Albermann et al., 2005) and SULT1C4 is involved in Dox disposition, catalyzing its sulfation (Luo et al., 2016), both anthracyclines and some of their predicted metabolites were *in silico* screened for their interaction with these targets. Docking results indicate that Dox had stronger predicted binding to PXR than all the other compounds, whereas only Dox, M15, M65, and M109 could bind to SULT. This suggests a better profile of Sdox against efflux transporters and SULT, compared to Dox and its metabolites.

Pharmacodynamics is the quantitative study of the relationship between drug exposure and pharmacological (desired) or toxicological (unwanted) responses.

In recent years, several efforts were made to ensure extensive identification of relevant cellular targets for a given drug both at the experimental (Bendels et al., 2019) and the computational level (Agamah et al., 2019; Bruno et al., 2019). *In silico* methods could help to anticipate for any given drug undesired interactions with other cellular targets, the so-called off-target effects (Rao et al., 2019). The study of these unwanted interactions had been proposed as strategy for drug repurposing (Lim et al., 2016). The main limitations are related to the availability of the three-dimensional structure with high resolution of the biological target and to the computational capacity. In the effort to expand the possibilities offered by this *in silico* approach, a method to profile differences between a parent drug and its derivatives was envisioned. In this work, we explored for Dox and Sdox relevant differences in the interaction with potiental off-targets. For our pilot study, we selected a set of 85 common cancer targets that are available in PDB. The objective was not to identify all possible off-targets for the two compounds but to point out relevant dissimilarity that might aid to explain the observed experimental differences in the biological assays.

Our method predicts that, differently from Dox, Sdox targets preferentially several proteins that are important for cell cycle progression, such as CDK6, mediating the G1/S transition; CDK7, which is required for the G2/M transition; KIF11, involved in the spindle dynamics during mitosis; ROCK1, necessary for the positioning of centrosome during mitosis; and DNA Topo I, allowing the rejoining of DNA single strands. Moreover, Sdox targets several proteins involved in proliferation and survival pathways, often oncogenically mutated and activated in tumors, such as RET tyrosine kinase, PKC-ζ, PKC-α, and the pro-angiogenic receptor VEGFR2. We are aware that all these predicted interactions and their biological meaning must be further validated experimentally, but the present findings suggest a multitarget profile of Sdox that acts simultaneously on different crucial proteins driving tumor progression.

We found larger ΔDS values among Dox targets. Worth mentioning is the strongest predicted interaction of Dox with the Vinca binding site of *β*-tubulin (ΔDS = 7.957 kcal/mol). The cardiac toxicity of anthracyclines was reported to be a consequence of the disruption of microtubule organization in cardiac myocytes (Fromes et al., 1996). Thus, it is reasonable to hypothesize that Sdox could exhibit a lower cardiac toxicity. The toxicity studies performed in zebrafish embryos and that obtained by Bigagli et al. (2018) and Gazzano et al. (2019) agree with this hypothesis.

The docking score is only a broad approximation to the predicted binding energy (not attempted to correlate with experimental ligand–receptor affinity) and is only used to give clues to possible interactions and ranks among compounds, prompting the further use of experimentation on these. Further biological tests will be necessary to validate the relevance of the off-targets, here outlined, in the different effects induced by both anthracyclines. Notably, the predicted differences seem to correlate with the experimental results. To the best of our knowledge, this work represents the first attempt to run such approach and the premise to further explore the potency of the predictive off-targets method in a future dedicated work. Overall, the differential target profile suggests that Dox and Sdox are two pharmacodynamically distinct drugs.

Kv11.1 (hERG) K^+^ channels mediate the cardiac IKr current that acts as an important determinant of action potential repolarization in the human ventricle and of pacemaking activity in heart nodes (Vandenberg et al., 2012). hERG blockade or dysfunction, therefore, results in prolongation of the electrocardiogram QT interval, leading, in rare cases, to Torsade de Pointes, a polymorphic ventricular tachycardia that can degenerate into ventricular fibrillation and death (Antzelevitch, 2007). Indeed, hERG channel liability, along with hepatotoxicity, is the major reason for drug attrition during preclinical development, clinical trials, and post-marketing drug withdrawal (Valentin, 2010).

*In silico* results predicted and *in vitro* data demonstrated that Dox and Sdox did not affect hERG currents recorded in hERG-HEK293 recombinant cells by using the patch-clamp technique.

Furthermore, Sdox, differently from Dox, was not cytotoxic in H9C2 cardiomyocytes (Chegaev et al., 2016; Buondonno et al., 2019), since the presence of H_2_S prevented the increase in reactive oxygen species (ROS) (Chegaev et al., 2016). Moreover, Sdox did not show any evidence of cardiac toxicity in prostate cancer xenograft mice (Bigagli et al., 2018), being the left ventricular wall thickness of mice treated with Sdox significantly lower than that of Dox-treated mice and comparable to that measured in vehicle treated animals. Additionally, Sdox displayed the same cardiotoxicity profile of Caelyx^®^ in osteosarcoma xenograft model (Gazzano et al., 2019).

Hepatic safety profile predicted either plausible or probable level of hepatotoxicity, for Sdox and Dox, respectively. Previous findings concerning liver toxicity indicated that Dox induces focal necrosis, hepatocytes vacuolation, degeneration of hepatocyte cords, and bile duct hyperplasia, mainly due to ROS generation during its hepatic metabolism, resulting in imbalanced redox potential leading to oxidative stress, reduced levels of antioxidant enzymes, apoptosis, inflammation, and mitochondrial dysfunction (Prasanna et al., 2020). In both hepatic cell-based models used here, Dox decreased cell viability and increased necrosis and oxidative stress, while Sdox was less cytotoxic and caused less oxidative damage.

These experimental findings were in agreement with previous works showing that Sdox did not display signs of liver toxicity, according to the hematochemical parameters (aspartate aminotransferase, alanine aminotransferase, alkaline phosphatase, and creatine phosphokinase) in osteosarcoma xenograft (Gazzano et al., 2019).

Several studies have already assessed the effects of Dox on zebrafish embryo development (Yang et al., 2011; Chang et al., 2014; Han et al., 2015; Cavalcante et al., 2021), and its toxicity was demonstrated at different developmental stages (Chang et al., 2014).

The results obtained in the present work demonstrated that unlike Dox, Sdox did not reduce the number of live embryos at 72 hpf.

It is known that the chorion can act as a selective barrier for some compounds to reach the embryo (Nishimura et al., 2016), but our results showed that this was not the case for both compounds.

The zebrafish expresses ABCB4 and ABCB5 transporters that are structurally very similar to mammalian P-gp (ABCB1); in particular, ABCB4 is responsible for embryo resistance to P-gp substrates (Fischer et al., 2013). However, Dox, a P-gp substrate, has no major effect on the zebrafish ABCB4 ATPase activity when applied at concentrations up to 100 µM [67].

At present, the interaction of Sdox with ATP-binding cassette membrane transporters such as ABCB4 and ABCB5, which are constitutively expressed in various tissues during early zebrafish embryo development, is unknown (Fischer et al., 2013).

*In vitro*, Sdox displayed a much lower Rf in SW1573 cells overexpressing P-gp as compared to Dox, indicating a different interaction pattern with this membrane transporter, either as a weak P-gp substrate or indirectly modifying its function. Therefore, it is conceivable to speculate that Sdox’s direct interaction with zebrafish ABCB4 transporter is also not prominent: further studies, however, are needed to clarify this issue.

The addition of H_2_S to Dox treatment in rats ameliorates its cardiotoxic effects by inhibiting oxidative stress, reducing inflammation, and suppressing apoptosis (Li et al., 2021). As Sdox treatment is accompanied by a reduced oxidative stress compared to Dox, the lack of toxic effects of the drug in zebrafish is likely due to the same mechanisms.

## Conclusion

Dox, discovered in the late 1960s, still represents the mainstay for the treatment of numerous solid and hematological malignancies, despite its therapeutic value being hampered by cross-resistance towards different anticancer drugs and severe dose-dependent cardiotoxicity.

Recent studies revealed that Sdox, a novel H_2_S-releasing Dox, besides being effective in several preclinical Dox-resistant tumor models, is also devoid of cardiotoxic effects.

This study aimed to further characterize Sdox integrating a multi- and trans-disciplinary approach.

*In silico* profiling suggested that Sdox possesses higher lipophilicity and lower solubility compared to Dox, and the off-targets prediction indicated that the proteins CDK6, MAPK 8, DNA Topo I, and PKC-ζ represent preferential targets for Sdox, whereas Dox was anticipated to bind preferentially the proteins *β*-tubulin (VBS), TNKS, GSK-3 β, and CCND3.

*In vitro* studies demonstrate that Sdox is a substrate with lower affinity for P-gp; it is less hepatotoxic and causes less oxidative damage than Dox.

Unlike Dox, it did not affect the percentage of Zebrafish live embryos at 72 hpf.

Although we cannot infer any conclusion about the clinical profile of Sdox, taken together, the *in silico*, *in vitro*, and *in vivo* findings demonstrate that it displays a higher efficacy against Pgp-positive cells, different selectivity towards cancer targets, and a more favorable ADME/toxicity profile than Dox, thus representing a significant advancement in the treatment of Dox-resistant/Pgp-overexpressing tumors.

## Data Availability

The original contributions presented in the study are included in the article/Supplementary Material, further inquiries can be directed to the corresponding author.
