# Supplementary material for: A Comprehensive Evaluation of Sdox, a Promising H2S-Releasing Doxorubicin for the Treatment of Chemoresistant Tumors
Source: Front Pharmacol. 2022 Mar 7;13:831791. doi: 10.3389/fphar.2022.831791 (PMC8936434; doi:10.3389/fphar.2022.831791)
Supplement: Supplementary file 1 [file Table5.pdf]

Table S5: Structures of the metabolites shared by Dox and Sdox predicted by the knowledge-based expert system Meteor Nexus (v. 3.1.0, Meteor KB 2018 1.0.0, Lhasa Ltd., https://www.lhasalimited.org/) with the minimal level of likelihood "plausible". The corresponding numbers of metabolites for Dox and Sdox respectively (Dox_Sdox) are listed below the structure.

1

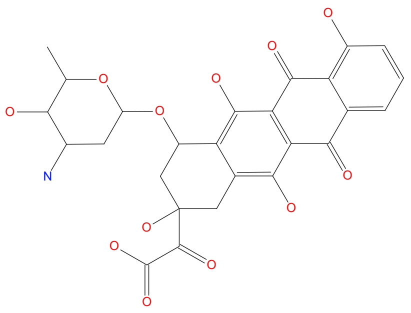

M33_M313

2

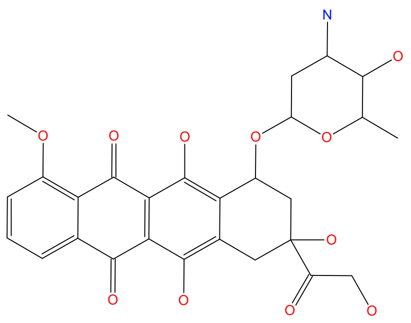

Dox_M16

3

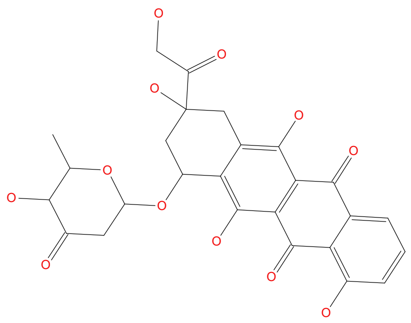

M34_M314

4

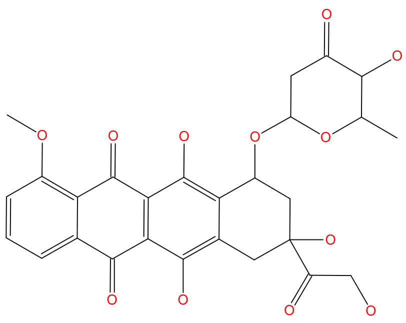

M9_M128

5

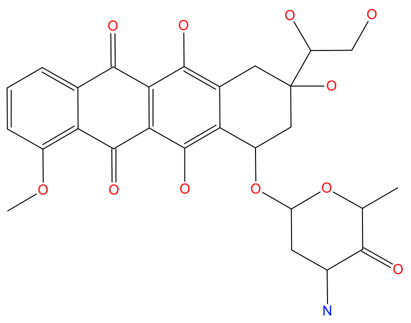

M16_M170

6

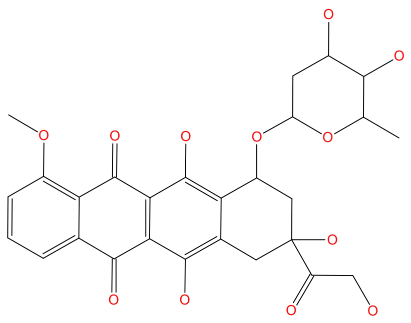

M50_M417

7

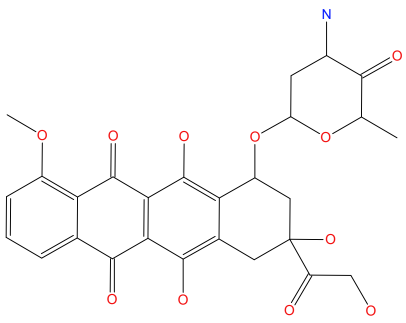

M1_M34

8

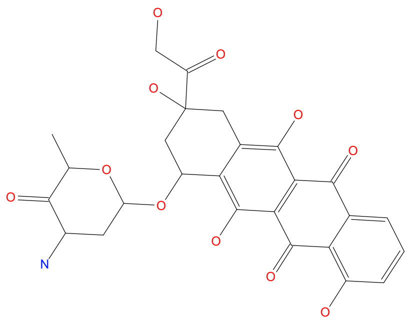

M15_M154

9

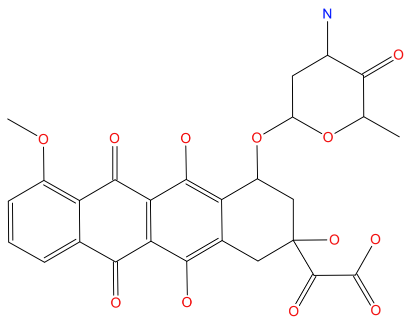

M17_M179

10

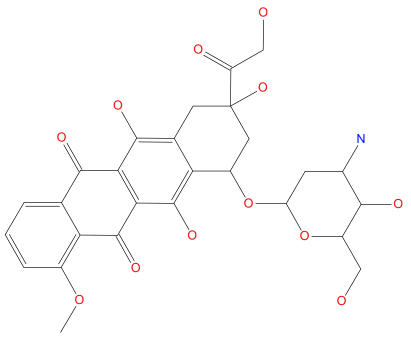

M2_M51

11

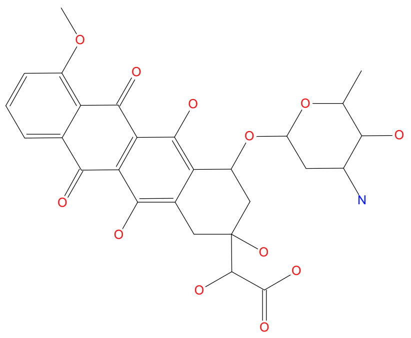

M39_M319

12

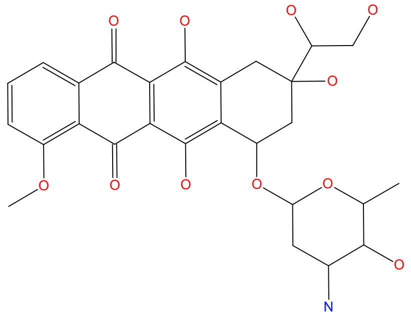

M7_M96

13

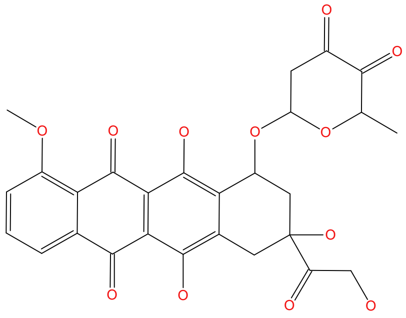

M18_M180

14

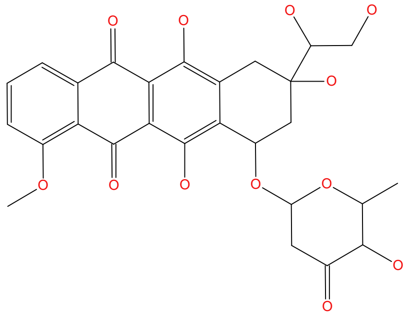

M40_M320

15

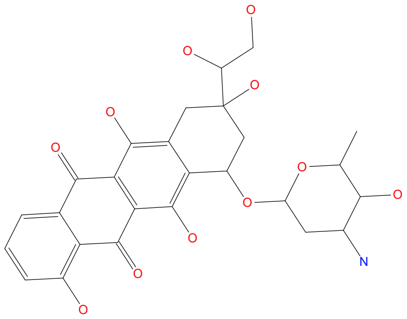

M32_M305

16

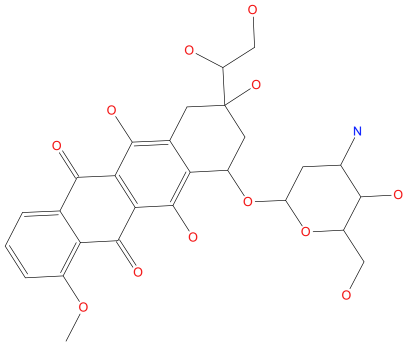

M24_M224

17

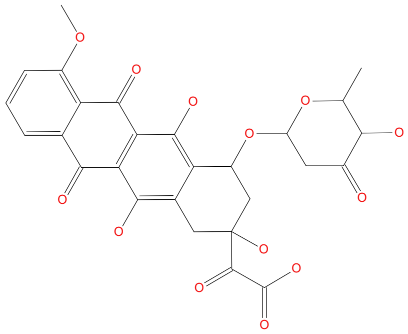

M45_M377

18

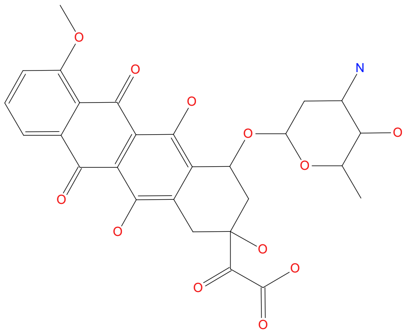

M8_M127

19

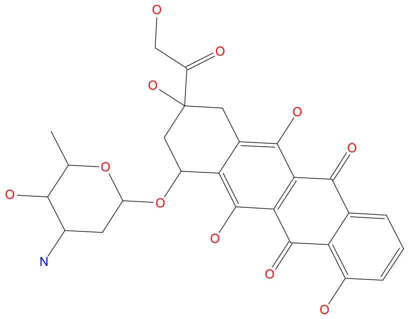

M6_M82
