# Supplementary material for: A Comprehensive Evaluation of Sdox, a Promising H2S-Releasing Doxorubicin for the Treatment of Chemoresistant Tumors
Source: Front Pharmacol. 2022 Mar 7;13:831791. doi: 10.3389/fphar.2022.831791 (PMC8936434; doi:10.3389/fphar.2022.831791)
Supplement: Supplementary file 3 [file Table4.pdf]

Table S4. Structures of Sdox metabolites predicted by the knowledge-based expert system Meteor Nexus (v. 3.1.0, Meteor KB 2018 1.0.0, Lhasa Ltd., https://www.lhasalimited.org/) with the minimal level of likelihood "plausible". The numbers of metabolites are listed below the structures (see also Table S2); 0 codes for metabolites that appear in the metabolic tree of Sdox only and 1 codes for metabolites that appear in the metabolic trees of both, Dox and Sdox (see Table S5).

1

Enzyme: ADH

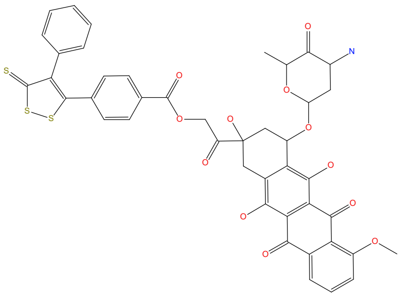

M1

0

2

Enzyme: CYP450

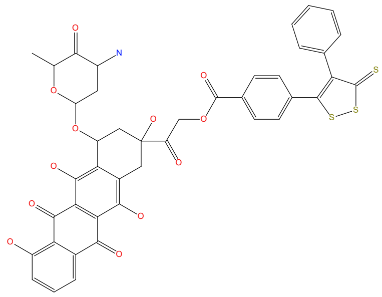

M31

0

3

Enzyme: Hydrolase

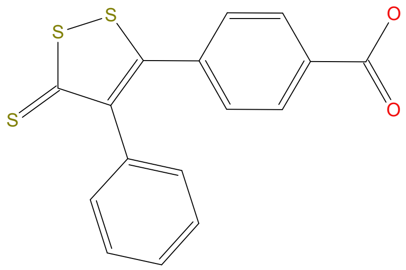

M15

0

4

Enzyme: Hydrolase

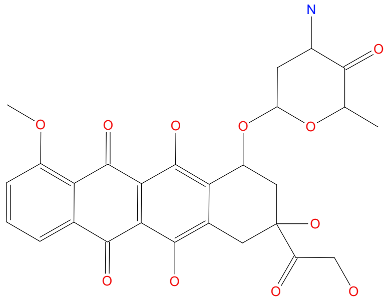

M34

1

5

Enzyme: CYP450

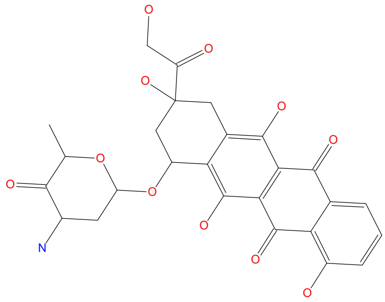

M154

1

6

Enzyme: ADH

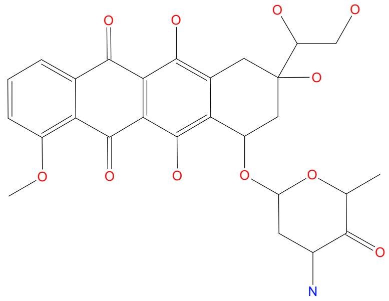

M170

1

7

Enzyme: ADH

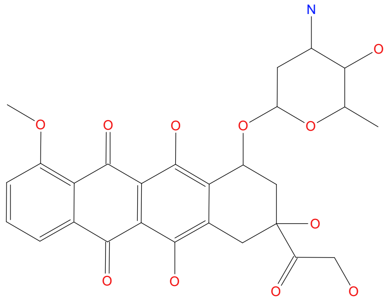

M16

1

8

Enzyme: ADH

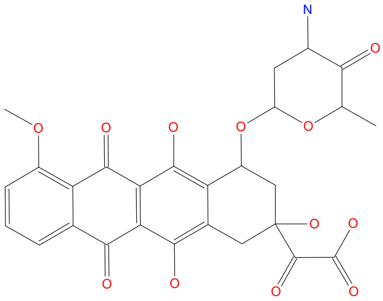

M179

1

9

Enzyme: ADH

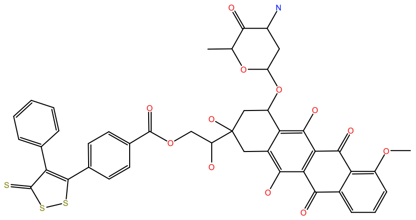

M32

0

10

Enzyme: ADH

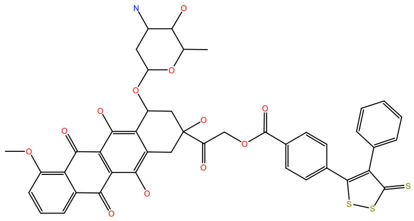

Sdox

0

11

Enzyme: CYP450

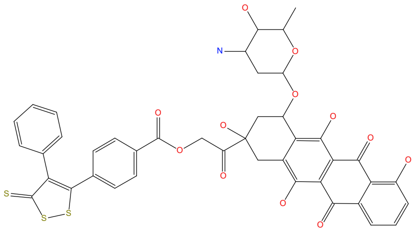

M12

0

12

Enzyme: ADH

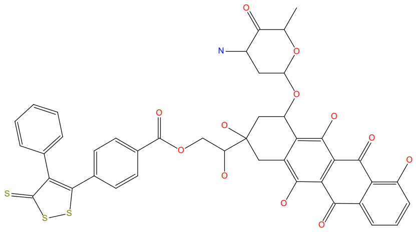

M152

0

13

Enzyme: Hydrolase

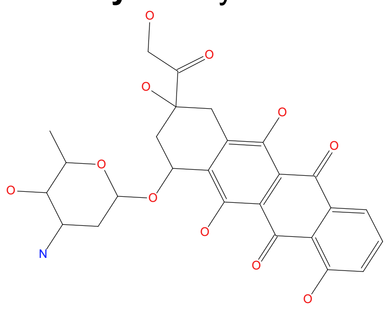

M82

1

14

Enzyme: ADH

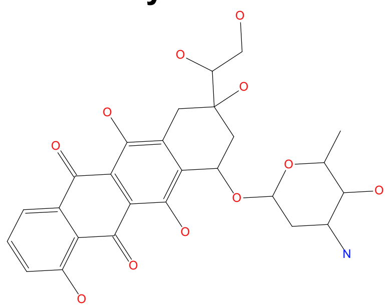

M305

1

15

Enzyme: ADH

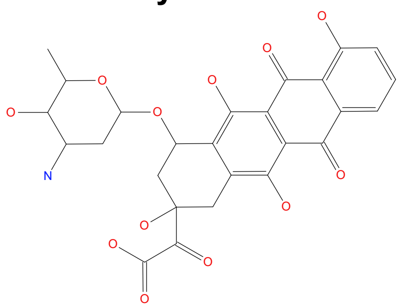

M313

1

16

Enzyme: ADH

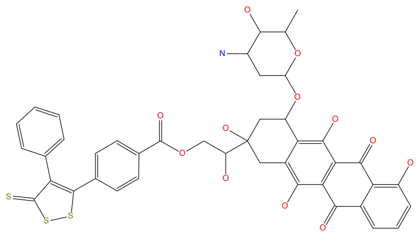

M80

0

17

Enzyme: ADH

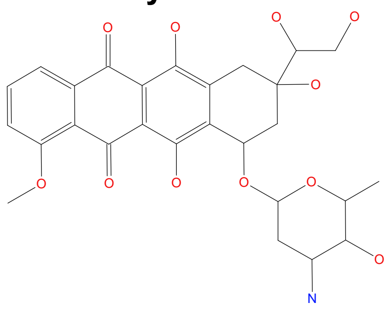

M96

1

18

Enzyme: ADH

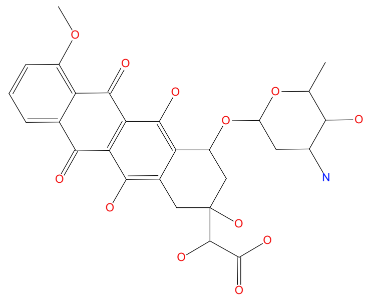

M319

1

19

Enzyme: ADH

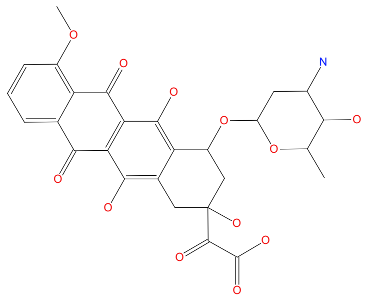

M127

1

20

Enzyme: CYP450

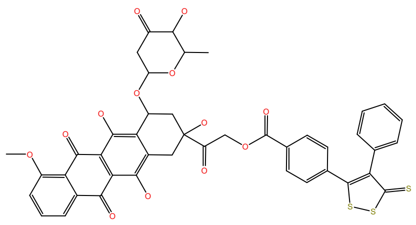

M17

0

21

Enzyme: ADH

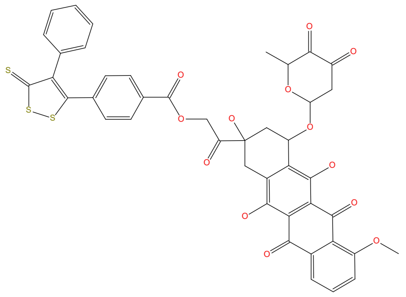

M35

0

22

Enzyme: CYP450

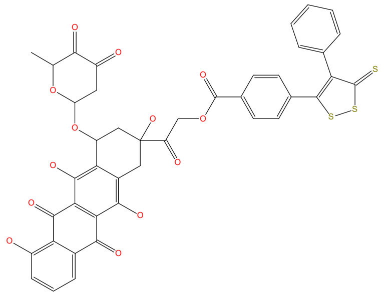

M155

0

23

Enzyme: Hydrolase

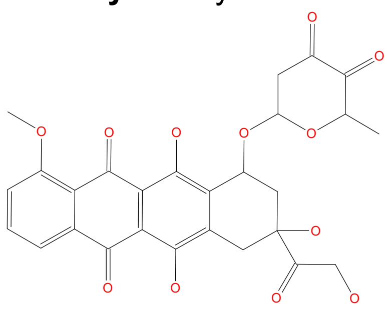

M180

1

24

Enzyme: ADH

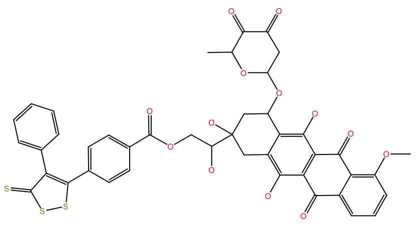

M171

0

25

Enzyme: CYP450

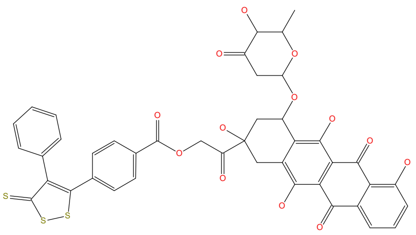

M83

0

26

Enzyme: Hydrolase

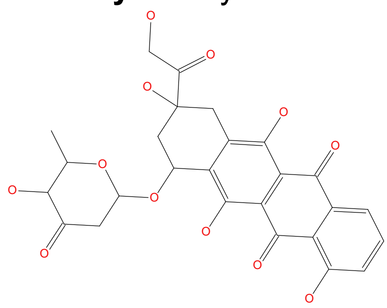

M314

1

27

Enzyme: ADH

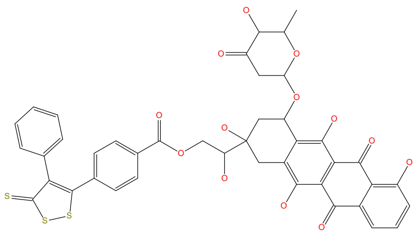

M306

0

28

Enzyme: ADH

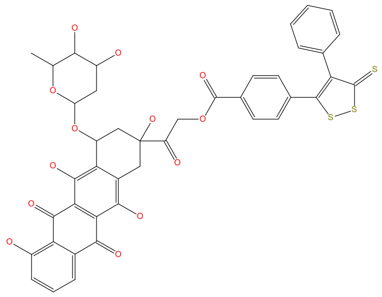

M398

0

29

Enzyme: Hydrolase

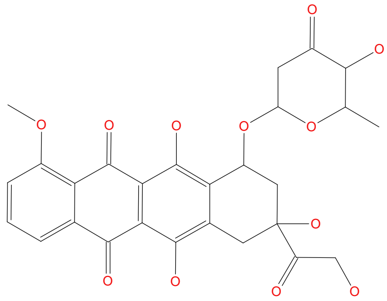

M128

1

30

Enzyme: ADH

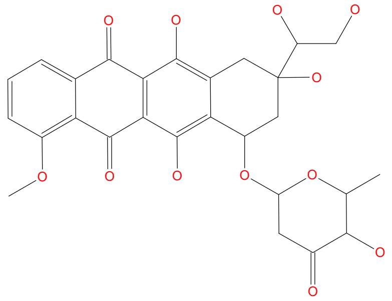

M320

1

31

Enzyme: ADH

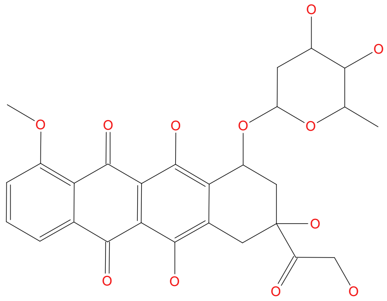

M417

1

32

Enzyme: ADH

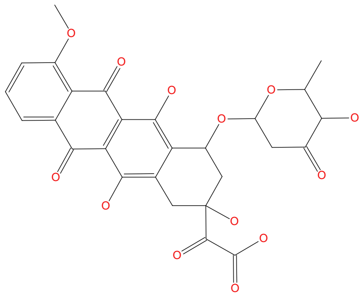

M377

1

33

Enzyme: ADH

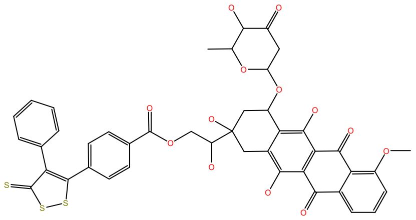

M97

0

34

Enzyme: ADH

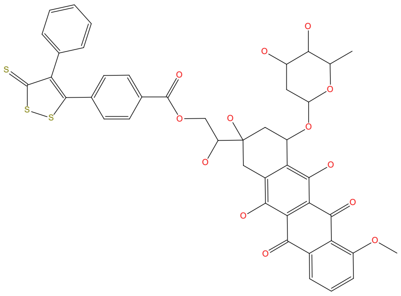

M410

0

35

Enzyme: ADH

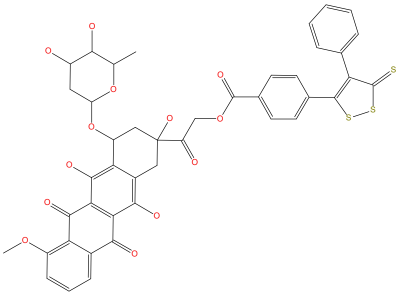

M137

0

36

Enzyme: ADH

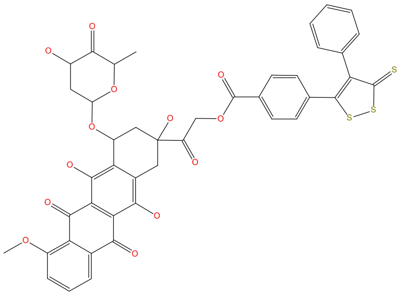

M420

0

37

Enzyme: CYP450

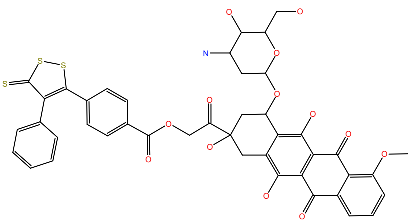

M2

0

38

Enzyme: ADH

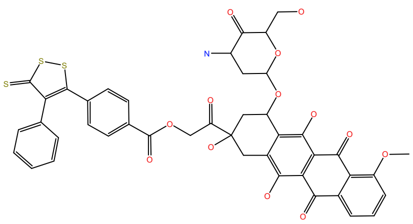

M21

0

39

Enzyme: CYP450

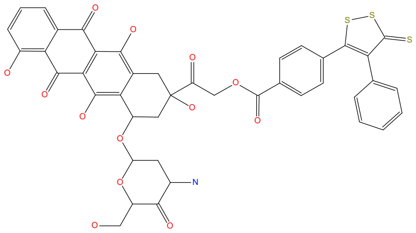

M142

0

40

Enzyme: Hydrolase

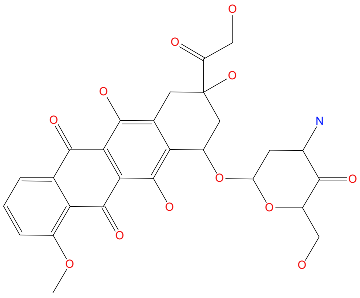

M175

0

41

Enzyme: ADH

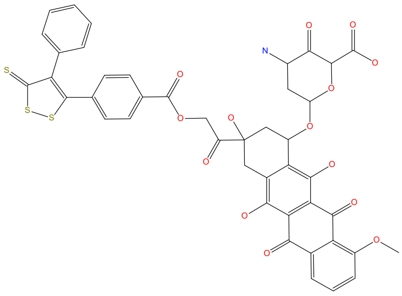

M192

0

42

Enzyme: ADH

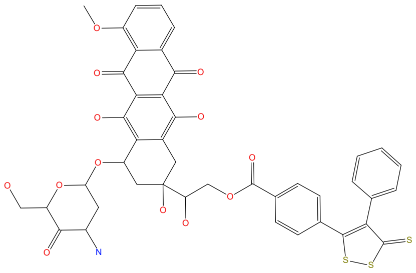

M159

0

43

Enzyme: CYP450

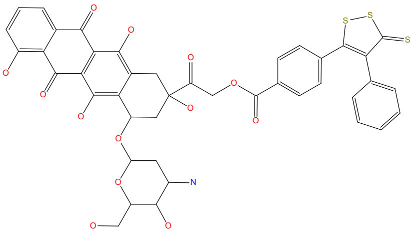

M48

0

44

Enzyme: Hydrolase

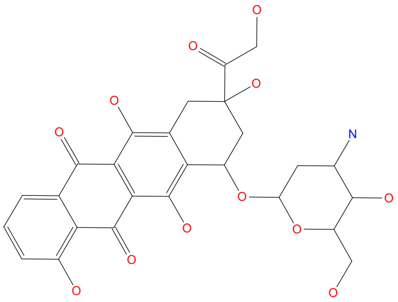

M208

0

45

Enzyme: ADH

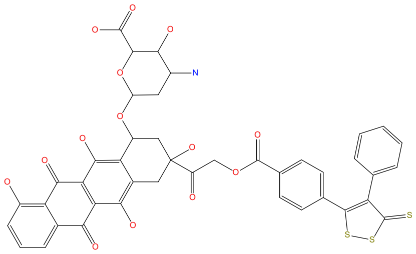

M209

0

46

Enzyme: ADH

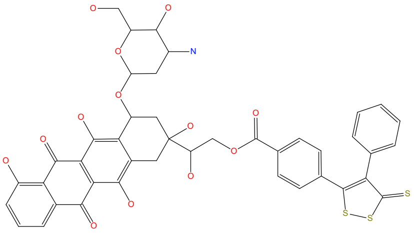

M206

0

47

Enzyme: Hydrolase

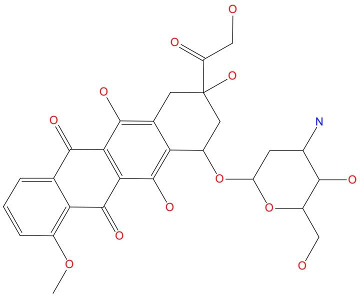

M51

1

48

Enzyme: ADH

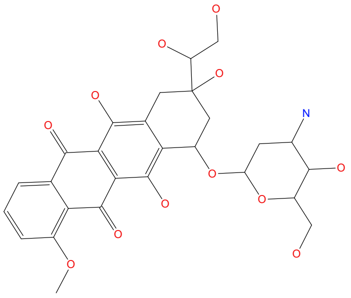

M224

1

49

Enzyme: ADH

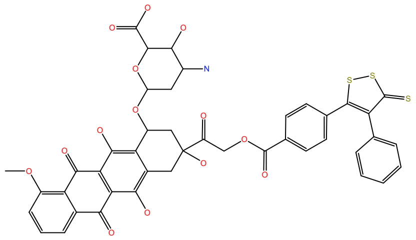

M52

0

50

Enzyme: ADH

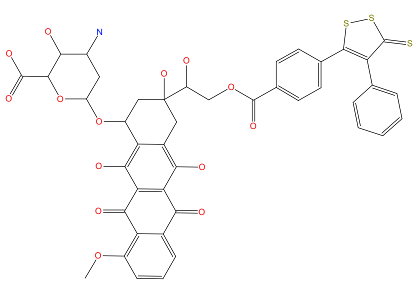

M225

0

51

Enzyme: Hydrolase

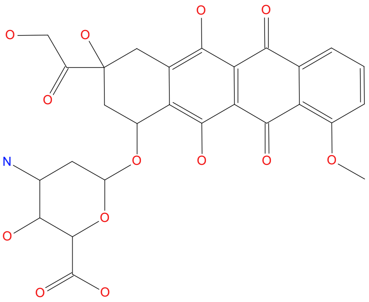

M234

0

52

Enzyme: ADH

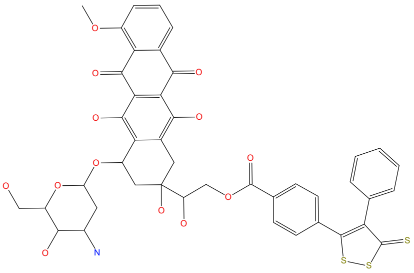

M49

0

53

Enzyme: CYP450

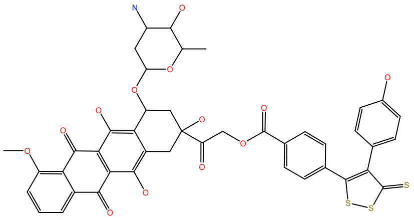

M5

0

54

Enzyme: ADH

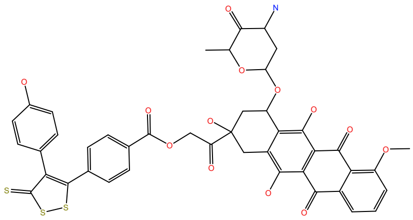

M24

0

55

Enzyme: CYP450

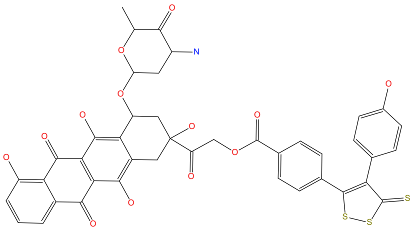

M145

0

56

Enzyme: Hydrolase

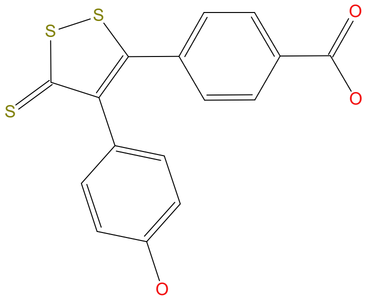

M65

0

57

Enzyme: ADH

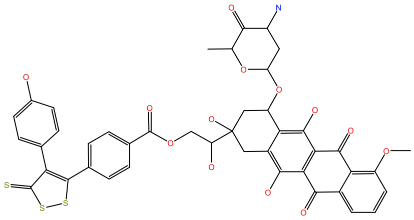

M162

0

58

Enzyme: CYP450

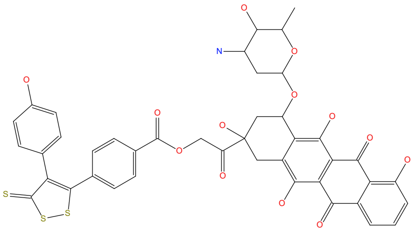

M62

0

59

Enzyme: ADH

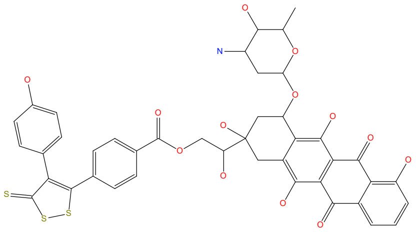

M268

0

60

Enzyme: ADH

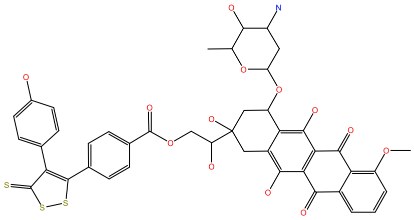

M63

0

61

Enzyme: ADH

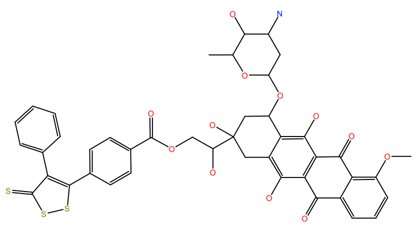

M13

0

62

Enzyme: GSR

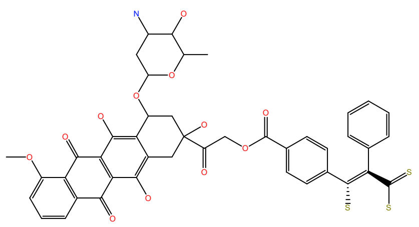

M14

0

63

Enzyme: ADH

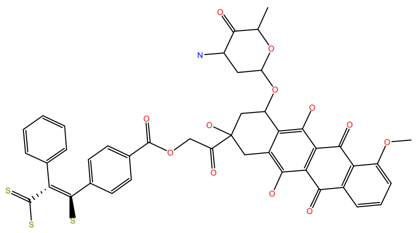

M33

0

64

Enzyme: CYP450

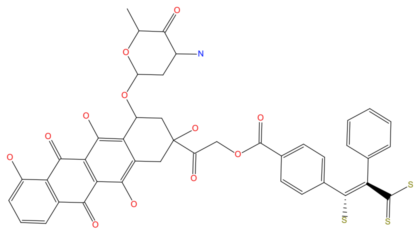

M153

0

65

Enzyme: Hydrolase

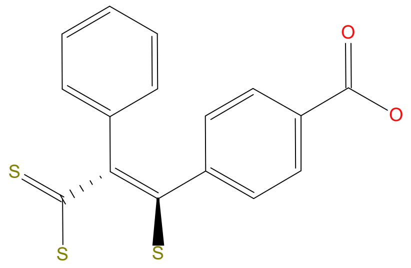

M109

0

66

Enzyme: ADH

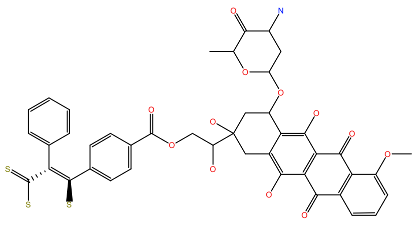

M169

0

67

Enzyme: CYP450

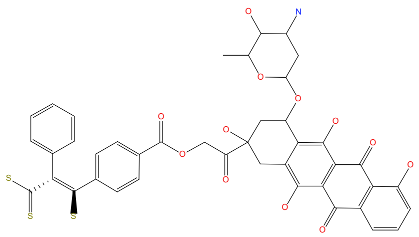

M81

0

68

Enzyme: ADH

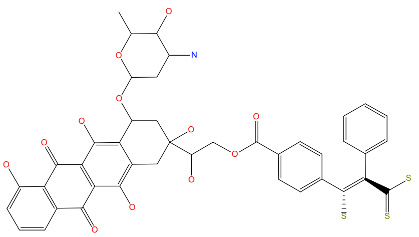

M304

0

69

Enzyme: ADH

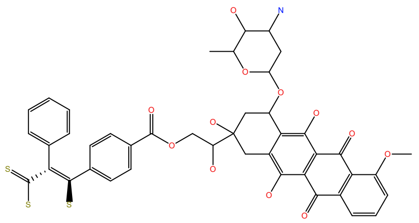

M95

0
