# Supplementary material for: A Comprehensive Evaluation of Sdox, a Promising H2S-Releasing Doxorubicin for the Treatment of Chemoresistant Tumors
Source: Front Pharmacol. 2022 Mar 7;13:831791. doi: 10.3389/fphar.2022.831791 (PMC8936434; doi:10.3389/fphar.2022.831791)
Supplement: Supplementary file 5 [file Table3.pdf]

Table S3. Structures of Dox metabolites predicted by the knowledge-based expert system Meteor Nexus (v. 3.1.0, Meteor KB 2018 1.0.0, Lhasa Ltd., https://www.lhasalimited.org/) with the minimal level of likelihood "plausible". The numbers of metabolites are listed below the structures (see also Table S1); 0 codes for metabolites that appear in the metabolic tree of Dox only and 1 codes for metabolites that appear in the metabolic trees of both, Dox and Sdox (see Table S5).

**Enzyme:** ADH<br>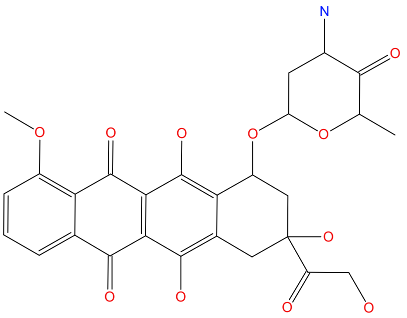<br>M1<br>1	**Enzyme:** CYP450<br>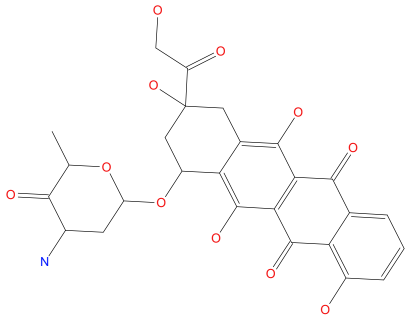<br>M15<br>1	**Enzyme:** ADH<br>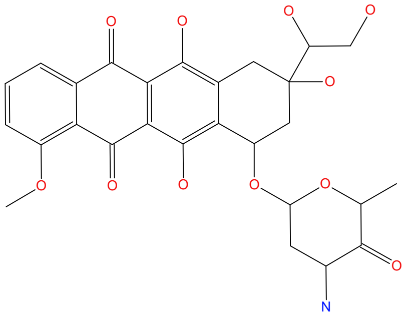<br>M16<br>1
**Enzyme:** ADH<br>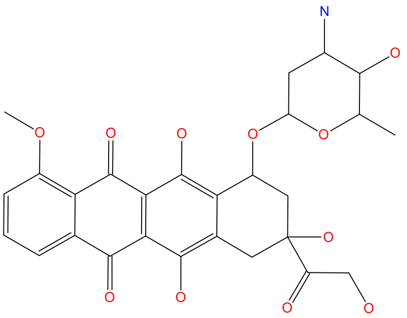<br>Dox<br>1	**Enzyme:** ADH<br>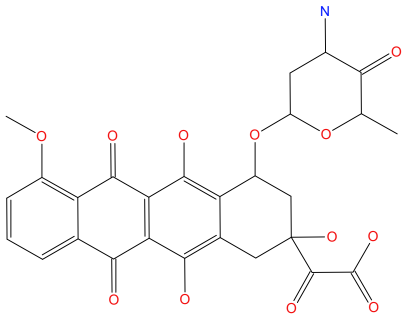<br>M17<br>1	**Enzyme:** ADH<br>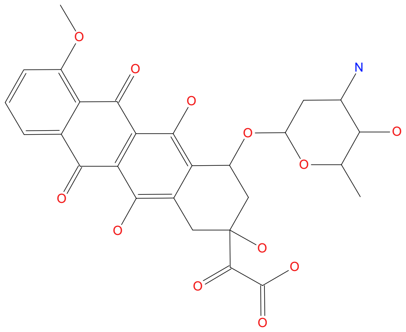<br>M8<br>1
**Enzyme:** CYP450<br>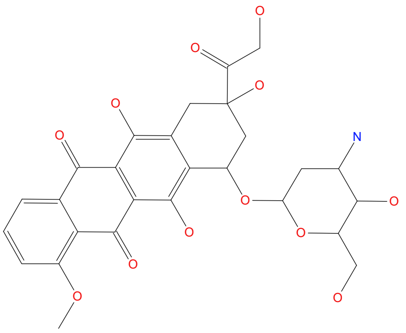<br>M2<br>1	**Enzyme:** ADH<br>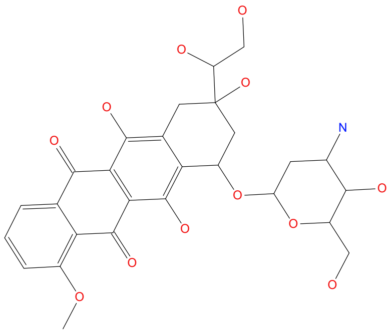<br>M24<br>1	**Enzyme:** ADH<br>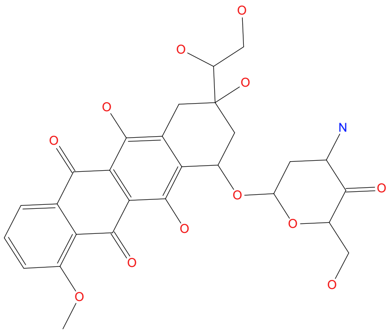<br>M61<br>0
**Enzyme:** CYP450<br>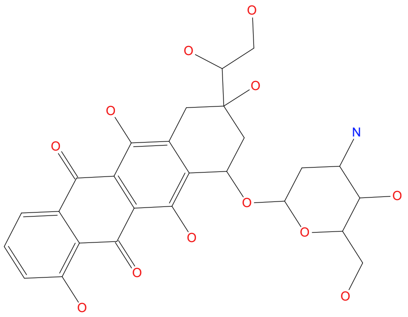<br>M78<br>0	**Enzyme:** ADH<br>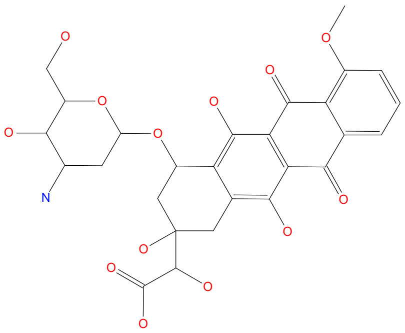<br>M79<br>0	**Enzyme:** ADH<br>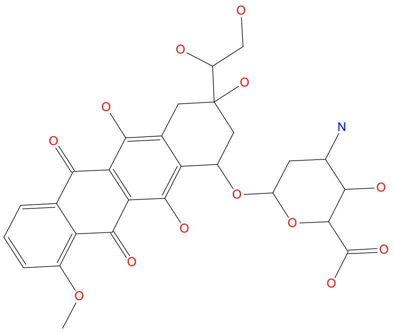<br>M80<br>0

Enzyme: CYP450

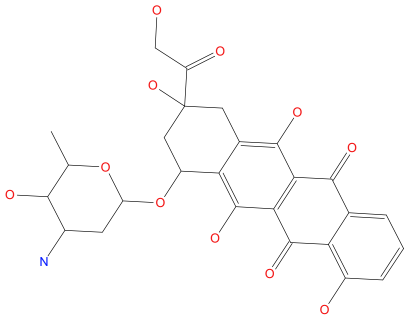

M6

1

Enzyme: ADH

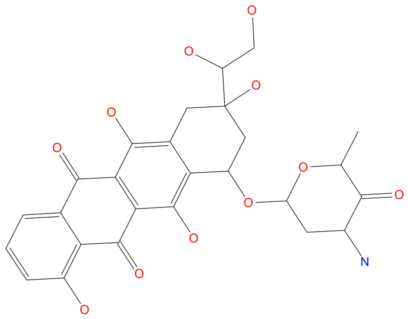

M57

0

Enzyme: ADH

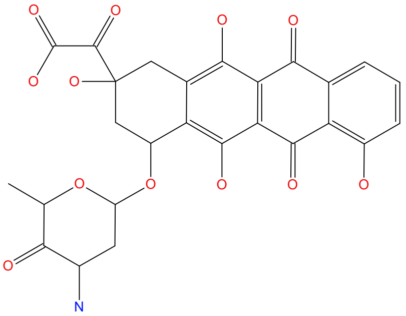

M58

0

Enzyme: ADH

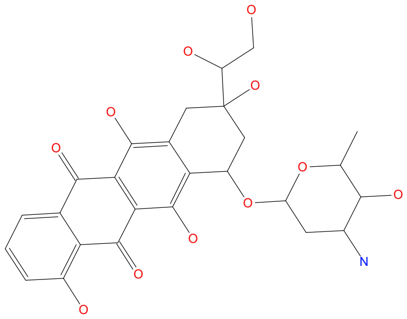

M32

1

Enzyme: ADH

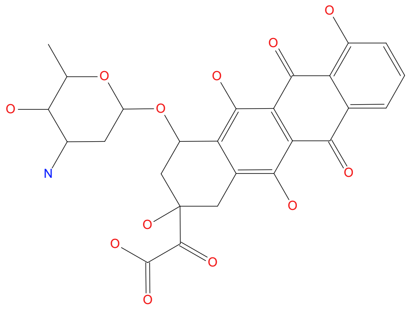

M33

1

Enzyme: ADH

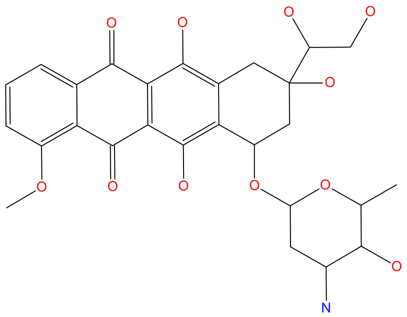

M7

1

Enzyme: ADH

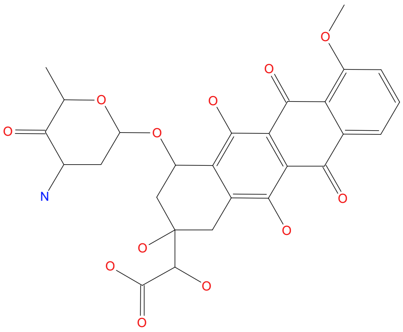

M65

0

Enzyme: ADH

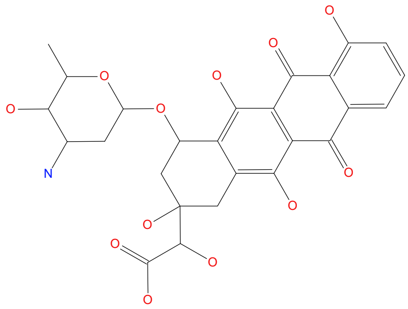

M86

0

Enzyme: ADH

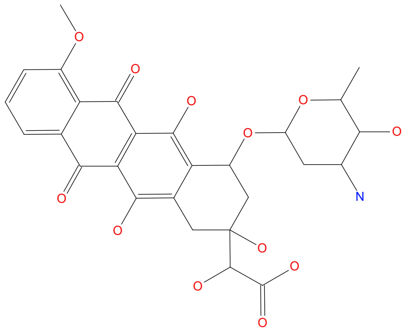

M39

1

Enzyme: CYP450

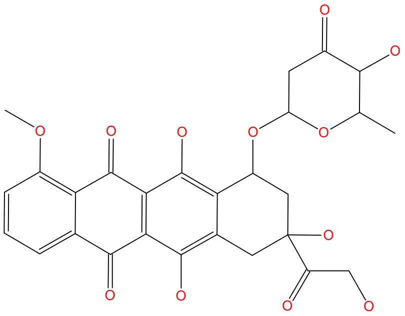

M9

1

Enzyme: ADH

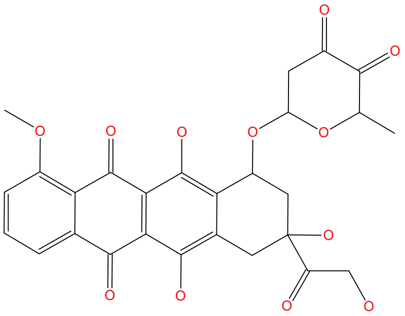

M18

1

Enzyme: CYP450

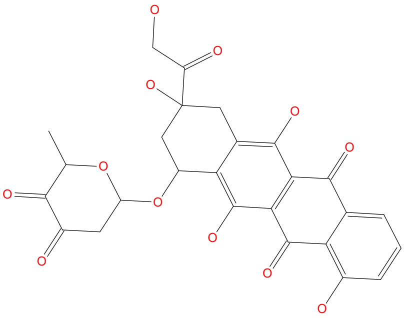

M59

0

Enzyme: ADH

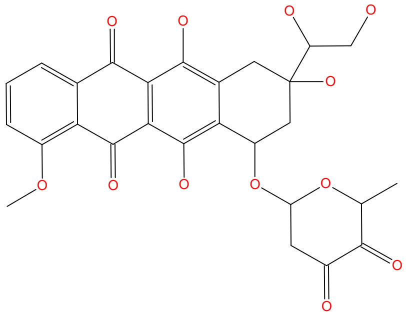

M66

0

Enzyme: ADH

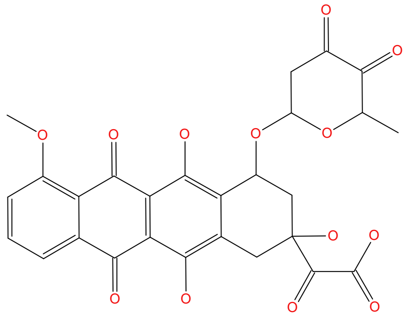

M72

0

Enzyme: CYP450

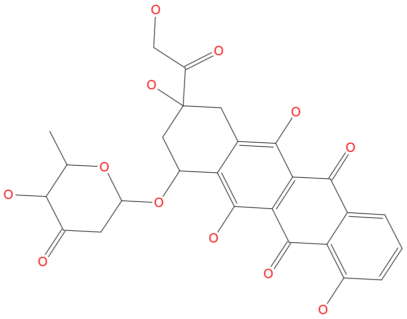

M34

1

Enzyme: ADH

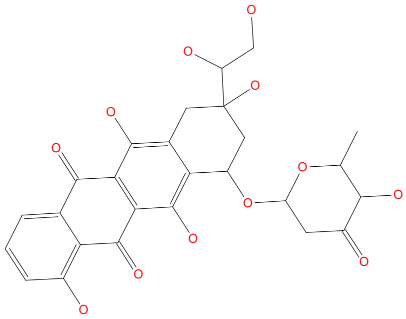

M87

0

Enzyme: ADH

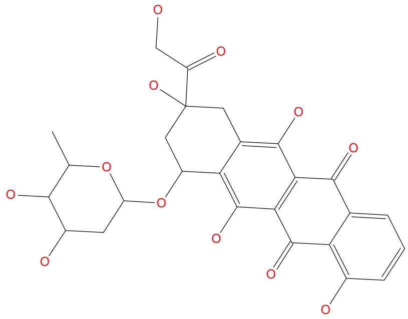

M109

0

Enzyme: ADH

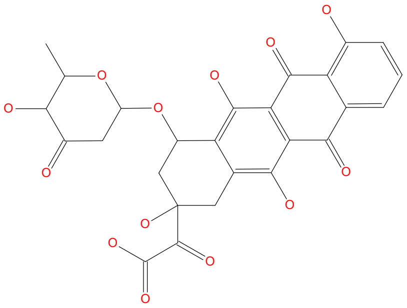

M93

0

Enzyme: ADH

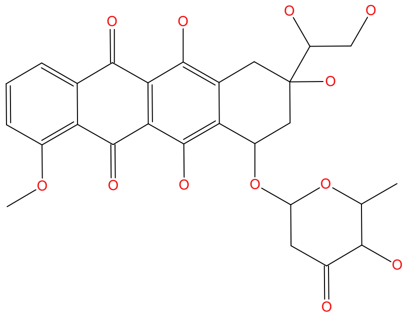

M40

1

Enzyme: ADH

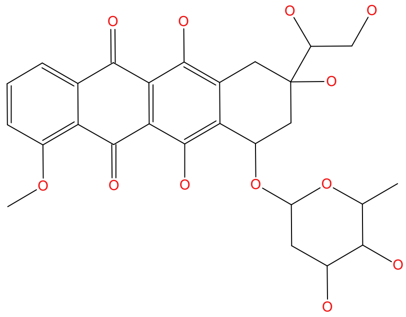

M114

0

Enzyme: ADH

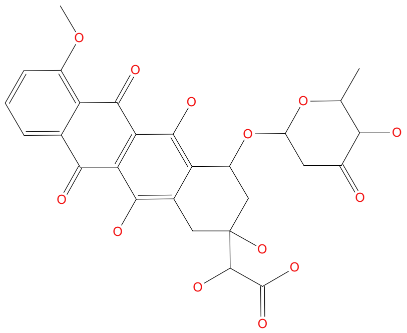

M99

0

Enzyme: ADH

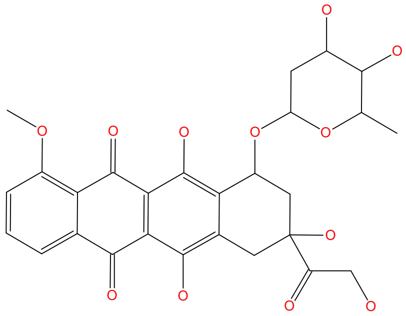

M50

1

Enzyme: ADH

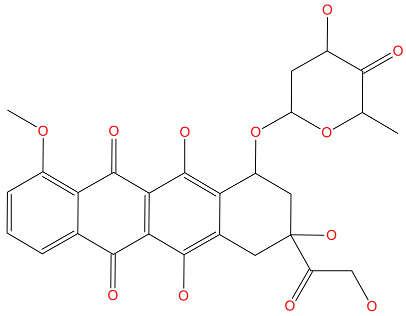

M124

0

Enzyme: ADH

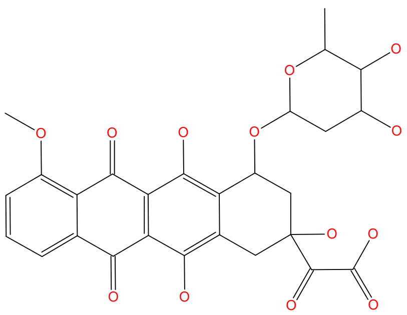

M120

0

Enzyme: ADH

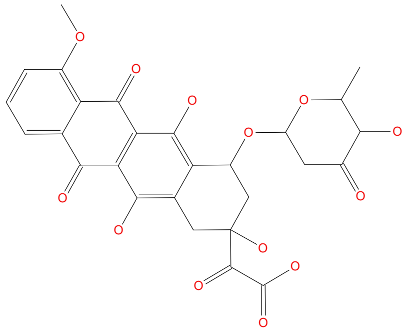

M45

1
