# Supplementary material for: A Comprehensive Evaluation of Sdox, a Promising H2S-Releasing Doxorubicin for the Treatment of Chemoresistant Tumors
Source: Front Pharmacol. 2022 Mar 7;13:831791. doi: 10.3389/fphar.2022.831791 (PMC8936434; doi:10.3389/fphar.2022.831791)
Supplement: Supplementary file 6 [file Table6.docx]

**Supplementary Table 6. Drug-protein interactions obtained for Sdox and Dox**

| Target | PDB ID | DS (kcal/mol) | | δDS  (kcal/mol) |
| --- | --- | --- | --- | --- |
|  |  | **Sdox** | **Dox** |  |
| CDK6 | 2euf | -11,344 | -8,119 | -3,225 |
| MAPK 8 | 2xrw | -9,667 | -7,096 | -2,571 |
| DNA topoisomerase 1 | 1rr8 | -9,358 | -6,880 | -2,478 |
| PKC theta | 2jed | -10,017 | -7,897 | -2,120 |
| VEGFR2 | 1y6a | -9,843 | -8,055 | -1,789 |
| Aurora Kinase B | 4af3 | -8,335 | -7,280 | -1,055 |
| ROCK1 | 2etk | -9,293 | -8,245 | -1,048 |
| KIF11 | 3wpn | -9,500 | -8,489 | -1,011 |
| CDK7 | 1ua2 | -9,132 | -8,265 | -0,867 |
| TNF-alpha | 2az5 | -7,589 | -6,736 | -0,853 |
| CDK1 | 4yc6 | -7,538 | -6,912 | -0,626 |
| mTOR | 4jsp | -8,300 | -7,697 | -0,604 |
| Hepatocyte growth factor receptor | 1r0p | -8,659 | -8,099 | -0,560 |
| RET | 2ivu | -9,115 | -8,572 | -0,543 |
| Mast/stem cell growth factor receptor | 3g0e | -8,763 | -8,546 | -0,217 |
| B-Raf | 3d4q | -8,534 | -8,326 | -0,208 |
| SH2 | 1o49 | -5,896 | -5,808 | -0,088 |
| Phosphatidylinositol-4,5-bisphosphate 3-kinase | 3hhm | -7,788 | -7,732 | -0,056 |
| ITK/TSK | 3mj2 | -8,035 | -8,008 | -0,027 |
| PAK 1 | 2hy8 | -8,179 | -8,161 | -0,018 |
| DNA topoisomerase 2-alpha | 4r1f | -7,055 | -7,041 | -0,014 |
| PKC alfa | 3iw4 | -8,831 | -8,849 | 0,018 |
| Bcl-2 | 2o21 | -6,821 | -6,875 | 0,054 |
| IGF1R | 2oj9 | -7,573 | -7,696 | 0,123 |
| HSP 90-beta | 1uym | -8,623 | -8,751 | 0,128 |
| BMX | 3sxr | -7,788 | -7,918 | 0,130 |
| GSK3 | 2dfm | -6,915 | -7,145 | 0,230 |
| CDC25B | 4wh9 | -6,291 | -6,556 | 0,265 |
| SHP-1 | 3ps5 | -6,901 | -7,187 | 0,286 |
| Peptide deformylase, mitochondrial | 3g5k | -7,788 | -8,138 | 0,350 |
| Ribosomal protein S6 kinase alpha-3 | 4d9u | -7,539 | -7,890 | 0,351 |
| CDC25A | 1c25 | -4,648 | -5,126 | 0,478 |
| MAPK 2 | 1s9i | -7,935 | -8,419 | 0,484 |
| Src | 2bdf | -7,590 | -8,096 | 0,506 |
| MAPK 1 | 1di9 | -6,545 | -7,052 | 0,507 |
| erbB-2 | 3rcd | -7,113 | -7,740 | 0,627 |
| HDAC8 | 1t64 | -6,302 | -6,954 | 0,652 |
| FAK1 | 4q9s | -7,467 | -8,124 | 0,657 |
| PKC iota | 1zrz | -7,543 | -8,313 | 0,770 |
| FGF Receptor 1 | 1agw | -6,864 | -7,761 | 0,897 |
| MDM2 | 1rv1 | -5,473 | -6,467 | 0,994 |
| ALK tyrosine kinase receptor | 2xb7 | -6,872 | -7,876 | 1,004 |
| Chk1 | 2hy0 | -7,022 | -8,039 | 1,017 |
| Calpain small subunit 1 | 4phk | -5,140 | -6,222 | 1,082 |
| Pyruvate dehydrogenase kinase isozyme 1 | 2q8g | -5,643 | -6,792 | 1,149 |
| MAPK 12 | 1cm8 | -7,583 | -8,747 | 1,164 |
| PI3K-gamma | 3apc | -6,517 | -7,725 | 1,208 |
| DHFR | 1boz | -7,534 | -8,769 | 1,235 |
| BRD4 | 3mxf | -6,972 | -8,267 | 1,295 |
| SYK | 1xbb | -6,520 | -7,834 | 1,314 |
| Apopain | 1pau | -5,907 | -7,283 | 1,376 |
| CDC25C | 3op3 | -5,116 | -6,595 | 1,479 |
| PKC eta | 3txo | -6,792 | -8,334 | 1,542 |
| XIAP | 1tft | -5,318 | -6,921 | 1,603 |
| ABL1 | 2gqg | -6,122 | -7,740 | 1,618 |
| FAK1 | 2etm | -5,703 | -7,396 | 1,693 |
| RSK1 | 2z7s | -6,165 | -7,899 | 1,734 |
| HSP 90-alpha | 1uy6 | -6,929 | -8,725 | 1,796 |
| PLK1 | 2rku | -6,392 | -8,219 | 1,827 |
| Ribosomal protein S6 kinase alpha-5 | 3kn5 | -6,661 | -8,501 | 1,840 |
| CDK2 | 1e1x | -5,957 | -7,825 | 1,868 |
| MEK1 | 3dy7 | -6,086 | -8,165 | 2,079 |
| ABL2 | 3hmi | -4,874 | -7,050 | 2,176 |
| AKT1 | 3qkk | -6,373 | -8,581 | 2,208 |
| Aurora Kinase A | 1mq4 | -6,409 | -8,629 | 2,220 |
| Tubulin-CBS | 4o2b | -4,933 | -7,244 | 2,311 |
| RAC1 | 3sua | -4,148 | -6,483 | 2,335 |
| PARP1 | 2rcw | -7,099 | -9,445 | 2,346 |
| Wee1 | 3cqe | -5,839 | -8,266 | 2,427 |
| GSK-3 beta | 1q3w | -4,967 | -7,406 | 2,439 |
| Chk2 | 2xm9 | -5,721 | -8,320 | 2,599 |
| JAK2 | 2b7a | -7,042 | -9,764 | 2,722 |
| PTPN11 | 3o5x | -4,366 | -7,095 | 2,729 |
| PKC beta | 2i0e | -5,903 | -8,678 | 2,775 |
| MSK1 | 1vzo | -5,264 | -8,326 | 3,062 |
| BTK | 3gen | -5,126 | -8,309 | 3,183 |
| MAPK 11 | 3gp0 | -5,897 | -9,149 | 3,252 |
| EGFR | 1m17 | -5,039 | -8,448 | 3,409 |
| FLT3 | 4rt7 | -4,058 | -8,052 | 3,994 |
| GART | 1njs | -4,310 | -8,725 | 4,415 |
| Mcl-1 | 4zbi | -1,931 | -6,468 | 4,537 |
| CCND3 | 3g33 | -1,705 | -7,340 | 5,635 |
| GSK-3 beta | 1o6k | -5,610 | -11,537 | 5,927 |
| TNKS | 4w5s | -1,692 | -7,729 | 6,037 |
| Tubulin-VBS | 4o2b | -7,477 | -15,434 | 7,957 |
