# Supplementary material for: A Comprehensive Evaluation of Sdox, a Promising H2S-Releasing Doxorubicin for the Treatment of Chemoresistant Tumors
Source: Front Pharmacol. 2022 Mar 7;13:831791. doi: 10.3389/fphar.2022.831791 (PMC8936434; doi:10.3389/fphar.2022.831791)
Supplement: Supplementary file 7 [file Image1.pdf]

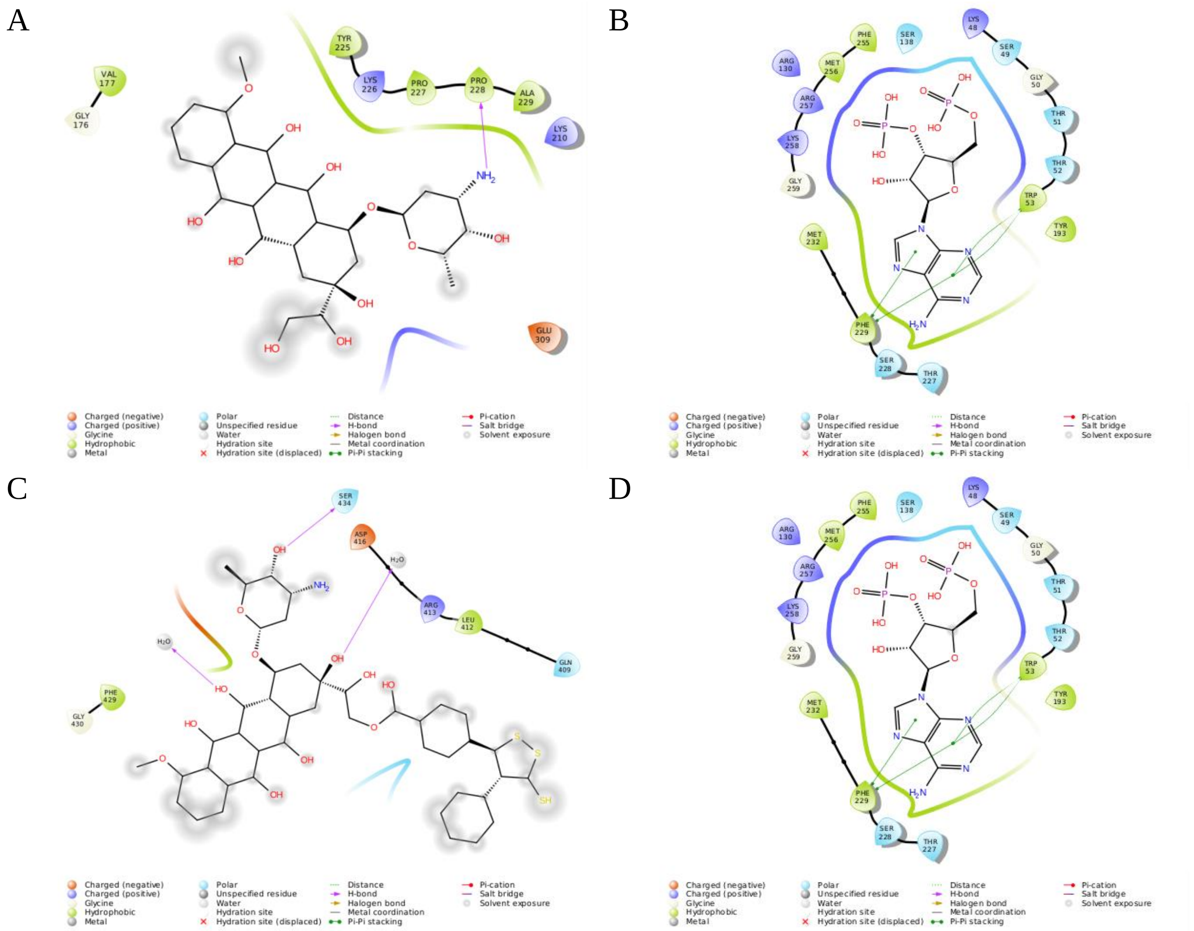

Supplementary Figure 1. Interactions for the complexes of: A) docked ligand Dox and PXR; B) docked ligand Dox and SULT; C) docked ligand Sdox and PXR; and D) docked ligand Sdox and SULT
